# Congenital neutropenia: diagnosis, molecular bases and patient management

**DOI:** 10.1186/1750-1172-6-26

**Published:** 2011-05-19

**Authors:** Jean Donadieu, Odile Fenneteau, Blandine Beaupain, Nizar Mahlaoui, Christine Bellanné Chantelot

**Affiliations:** 1 Service d'Hémato Oncologie Pédiatrique Registre des neutropénies congénitales AP-HP Hopital Trousseau 26 avenue du Dr Netter F 75012 Paris, France; 2 Laboratoire d'hématologie AP-HP Hôpital R Debré Boulevard Sérurier Paris, 75019, France; 3 Unité d'Immuno-Hématologie et Rhumatologie pédiatriques, et Centre de référence des déficits Immunitaires Héréditaires (CEREDIH), Groupe Hospitalier Necker-Enfants Malades, Assistance Publique-Hôpitaux de Paris, Paris 75015, France; 4 Département de Génétique, AP-HP Groupe Hospitalier Pitié- Salpêtrière, Université Pierre et Marie Curie, 47/83 Bd de l'Hôpital Bâtiment 10 Lapeyronie 75651 Paris Cedex 13, France

**Keywords:** Neutropenia, Childhood, G-CSF, Severe congenital neutropenia, Adverse effects, *ELANE*, *G6PC3*, Shwachman Diamond Syndrome, Review

## Abstract

The term congenital neutropenia encompasses a family of neutropenic disorders, both permanent and intermittent, severe (<0.5 G/l) or mild (between 0.5-1.5 G/l), which may also affect other organ systems such as the pancreas, central nervous system, heart, muscle and skin. Neutropenia can lead to life-threatening pyogenic infections, acute gingivostomatitis and chronic parodontal disease, and each successive infection may leave permanent sequelae. The risk of infection is roughly inversely proportional to the circulating polymorphonuclear neutrophil count and is particularly high at counts below 0.2 G/l.

When neutropenia is detected, an attempt should be made to establish the etiology, distinguishing between acquired forms (the most frequent, including post viral neutropenia and auto immune neutropenia) and congenital forms that may either be isolated or part of a complex genetic disease.

Except for ethnic neutropenia, which is a frequent but mild congenital form, probably with polygenic inheritance, all other forms of congenital neutropenia are extremely rare and have monogenic inheritance, which may be X-linked or autosomal, recessive or dominant.

About half the forms of congenital neutropenia with no extra-hematopoetic manifestations and normal adaptive immunity are due to neutrophil elastase (*ELANE*) mutations. Some patients have severe permanent neutropenia and frequent infections early in life, while others have mild intermittent neutropenia.

Congenital neutropenia may also be associated with a wide range of organ dysfunctions, as for example in Shwachman-Diamond syndrome (associated with pancreatic insufficiency) and glycogen storage disease type Ib (associated with a glycogen storage syndrome). So far, the molecular bases of 12 neutropenic disorders have been identified.

Treatment of severe chronic neutropenia should focus on prevention of infections. It includes antimicrobial prophylaxis, generally with trimethoprim-sulfamethoxazole, and also granulocyte-colony-stimulating factor (G-CSF). G-CSF has considerably improved these patients' outlook. It is usually well tolerated, but potential adverse effects include thrombocytopenia, glomerulonephritis, vasculitis and osteoporosis. Long-term treatment with G-CSF, especially at high doses, augments the spontaneous risk of leukemia in patients with congenital neutropenia.

## Background

Congenital neutropenia is characterized by chronic neutropenia due to a constitutional genetic defect. Since the early 1990s, and particularly during the last decade, the molecular bases of several entities have been discovered, leading to changes in the disease classification. Kostmann's syndrome is often considered as the paradigm of congenital neutropenia. This disorder, first described in a Swedish publication in 1950 [1], and subsequently in English in 1956 [2], has three main characteristics: profound neutropenia (<0.2 G/l) occurring during the first weeks of life, maturation arrest of granulopoiesis at the promyelocyte stage, and death due to bacterial infections (11 of the 14 initially reported patients died in their first year of life from bacterial infections). Nearly 50 years later, these patients' life expectancy routinely exceeds 20 years and the molecular basis of this entity has been identified [3]. It is now agreed that Kostmann's syndrome is accompanied, at least in forms due to mutations of one the two isoforms of *HAX1 *protein, those observed in the 'kostmann's pedigree', by neurological involvement (mental retardation and epilepsy) [4]. Thus, the "paradigm" of congenital neutropenia is a condition with early hematologic expression and later neurological involvement.

Knowledge of the molecular bases of other forms of congenital neutropenia has also modified the disease classification. Until the late 1990s, the literature distinguished cyclic neutropenia, associated with a regular pattern of change in the neutrophil count, typically every 21 days and showing autosomal dominant transmission [5], from permanent neutropenia (severe congenital neutropenia or Kostmann's syndrome). This distinction was made in publications based on the international registry of chronic neutropenia in the late 1990s [6,7], in which cyclic neutropenia was not included among the congenital neutropenias. In 1999, M. Horwitz, analyzing 13 pedigrees of patients with cyclic neutropenia, identified mutations in the neutrophil elastase (*ELANE*) gene [8]. Shortly afterwards the same team found that many patients with severe congenital neutropenia also had mutations of the *ELANE *gene [9] This pointed to a continuum between severe congenital neutropenia and cyclic neutropenia, and showed that both could be considered "congenital".

Another example of nosologic reclassification concerns the gluco-6-phosphatase molecular complex, which is defective in glycogen storage disease Ib and also in an entity associated with cutaneous involvement, cardiac arrhythmias and malformative uropathy but not with metabolic disorders [10]

## Definition: neutropenia and congenital neutropenia

### General definition

Neutropenia is defined as a reduction in the absolute number of neutrophils in the blood circulation. The standard hematologic examination is microscopic cell counting, which is necessary to confirm disorders identified by automated cell counters and especially to examine the cell morphology. Neutropenia is defined by a neutrophil count below 1.5 G/l in children over 1 year, and below 2 G/l in children aged between 2 and 12 months [11-13].

The number of neutrophils is elevated during the first two months of life. The count increases during the first 72 hours, followed by a gradual decrease until the age of two months. In term neonates the neutrophil count is reported to range from 12 G/l to 15 G/l, depending on the study. Labor lasting more than 12 hours is associated with higher counts, while prematurity (<32 weeks) is associated with lower counts. Neutropenia in newborns is therefore defined by a threshold higher as in adult at least 2.5 G/l neutrophils.

Neutropenia is said to be severe when below 0.5 G/l and chronic if it lasts more than 3 months, whether it is intermittent or permanent.

It is important to stress that the neutrophil count shows physiological fluctuations [14], in a chaotic and non random manner [15]. There are also nycthemeral and seasonal variations [16-18], which persist in pathologic situations. Thus, neutropenia should ideally be confirmed on three samples per week over a 6-week period.

Neutropenia is said to be permanent when present in all samples, intermittent if there are periods of spontaneous normalization, and cyclic if episodes occur about every 21 days (perfectly sinusoidal neutropenia with a 21-day cycle is almost never seen in practice).

Only one study has focused on the periodicity in patients with a diagnosis of "cyclic" neutropenia, based on serial counts [19]. Among 10 such patients, regular periods, of 18, 20 and 30 days, were found in only three cases. The same study also showed regular variations in patients with permanent neutropenia (severe congenital and idiopathic neutropenia). Thus, it is better to use the terms "permanent neutropenia" and "intermittent neutropenia", while bearing in mind that there is a continuum between the two extremes, as the pathological processes that lead to neutropenia affect both the period of variation and the depth of the nadir.

**Figure 1:**
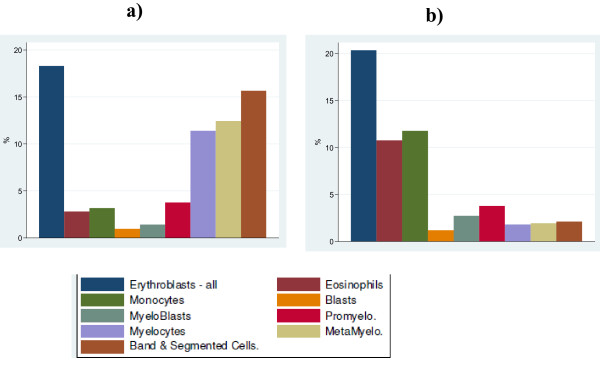
**Bone marrow smear differential count including % of the different granulocyte precursors**. a) normal bone marrow - a regular pyramid. b) patients with severe congenital neutropenia and *ELANE *mutation: bone marrow myeloid arrest at promyelocyte stage with eosinophilia

Neutropenia is said to be "central" when the bone marrow compartment is depleted, as shown by a deficiency in late maturation stages (especially <10% of mature neutrophils) and "peripheral" if bone marrow neutrophil maturation is normal (Figure 1).

Monocytosis, hypereosinophilia and polyclonal hypergammaglobulinemia are associated with neutropenia and are inversely proportional to its severity. A compensatory role of monocytes may explain the good clinical tolerance of some forms of profound constitutional neutropenia [20]

### Congenital neutropenia: an evolving definition

Close examination of the literature shows that the term "congenital neutropenia" is not used homogeneously [6,21-23]. One very restrictive definition reserves the term "congenital neutropenia" for severe forms not associated with immunological or extra-hematopoeitic abnormalities, while a broader definition includes all situations that comprise chronic neutropenia, with or without immunological or extra-hematopoeitic abnormalities. Thus, some authors but not others include glycogen storage disease Ib, Shwachman-Diamond syndrome, the WHIM syndrome, and Barth's disease in the definition of congenital neutropenia.

In this review, the term "congenital neutropenia" is not restricted to disorders in which neutropenia is the only phenotypic manifestation, but encompasses all 'congenital' disorders comprising neutropenia. We also consider "neutropenia" as a continuum, ranging from intermittent forms with various periods to permanent circulating neutrophil deficiency.

## Epidemiology

The data are currently limited, owing to confusion and overlapping case definitions. Exhaustive studies are rare, and few patient registries are available[24]. General epidemiological surveys of primary immune deficiencies do not take congenital neutropenia into account [25-27], with the exception of the Iranian study [28] and a recent French study [29]. In the Iranian study, 53 cases were recorded, for a prevalence of 0.77/10^6^. In the French registry-based study of a population of comparable size, 374 cases had been recorded in December 2006, giving a prevalence nearly 10 times higher ≈ 6.2/10^6^. Neither study included patients with idiopathic neutropenia.

In 2003, the International Neutropenia Registry [6] reported 731 cases, of which 238 were idiopathic, recruited in a far larger geographic area than in previous studies, including the USA, Canada, Australia and Europe (excluding France), for a population close to 700 million. The prevalence was 0.7 per million inhabitants or 1 per million inhabitants when idiopathic neutropenia was included.

There are probably no major differences across countries, and the minimal prevalence of congenital neutropenia appears to be 6 cases per million inhabitants, if we take into consideration the results from the French survey - the highest rate so far described. In the French registry, 30% of patients had *ELANE *neutropenia (20% severe congenital neutropenia and 10% cyclic neutropenia), 30% had Shwachman-Diamond syndrome, 5% had glycogen storage disease Ib, and 35% had other disorders (1 or 2% each). However, the distribution of the different forms was influenced by the patients' geographic origin (e.g. immigrants to western countries). Some mutations are also linked to the geographic origin (*HAX1 *in Kurdistan and Sweden, *G6PC3 *in Arameans, AP14 in Mennonites), while *ELANE*, *SBDS*, *SLC37A4 (*previously named G6PT1) and *CXCR4 *mutations appear to be universally distributed.

## Clinical description

### The consequences of neutropenia: infections

In vitro, the antibacterial activity of neutrophils can be represented by a simple dilution curve [30]. The risk of bacterial infection is more difficult to appreciate in vivo. Central neutropenia carries a far higher risk of bacterial and fungal infections than peripheral neutropenia. In central neutropenia the risk is low at counts above 1 G/l, increases moderately between 1 and 0.2 G/l and is very high below 0.2 G/l. The risk of infection also depends on the duration of neutropenia, with the risk of fungal infections increasing after several weeks. These data were obtained some 30 years ago in leukemic patients [31] and more recently in bone marrow graft recipients [32]. They correspond to the natural history of some constitutional forms of central neutropenia, especially that described by Kostmann [2,33], although this has not been confirmed by other authors [34]. The preferential sites of infection are highly variable. The most frequent are the skin and mucosae, the ENT region, and the lungs. Stomatologic disorders are almost always present after age two years in patients with profound central neutropenia, and are characterized by erosive, hemorrhagic and painful gingivitis associated with papules (aphthae-like oral furuncles) of the tongue and the cheek mucosa (Additional file 1, Figure S1 Plates #1 and #2) [35]. Diffuse gastrointestinal lesions are sometimes present, leading to abdominal pain and diarrhea, and sometimes mimicking Crohn's disease on radiological studies [36]. These lesions may also be related to bacterial enteritis. It should be remembered that the symptoms of such infections may be atypical in patients with profound neutropenia, with local inflammation, the absence of pus and a necrotic tendency. One particular aspect is ecthyma gangrenosum (infectious perianal ulceration). Bacterial infections are most frequent, and generally involve Staphylococcus aureus and epidermidis, streptococci, enterococci, pneumococci, Pseudomonas aeruginosa, and Gram-negative bacilli. Most fungal infections involve Candida or Aspergillus species.

### Extra-hematopoietic involvement

A variety of extra-hematopoietic involvement may be observed, contributing to the definition of several diseases or syndromes that will be examined in the Classification section, tables 1 and 2, and the Etiology/Classification section.

**Table 1:** Monogenic congenital neutropenia: Review of the known genes (2010)

Sub group of neutropenia	Disease name/ref	OMIM code	Main hematological features	Extra-hematopoeitic features	Inheritance	Gene localisation	Gene (alias)	Normal function of the gene
								

Congenital Neutropenia without extra hematopoeitic manifestations	Severe congenital neutropenia/Cyclic neutropenia [8,43]	202700 162800	Severe and permanent Maturation arrest Intermittent/cyclic with variable bone marrow features	No	Dominant	19q13.3	*ELANE*	Protease activity Antagonism with alpha 1 antitrypsin
	
	Severe congenital neutropenia Somatic mutation of CSF3R	202700	Permanent Maturation arrest Unresponsive to GCSF	No	No genetic inheritence	1p35-p34.3	*CSF3R*	transmembrane GCSF receptor/intracellular signalling

Congenital Neutropenia with innate or adaptive deficiency but no extrahematopoietic features	Severe congenital neutropenia [88]	202700	Permanent/severe or mild Sometimes maturation arrest	Internal ear (in mouse model) Lymphopenia	Dominant	1p22	*GFI1*	Transcription factor Regulation of oncoprotein
	
	Severe congenital neutropenia [89,92]	301000	Severe permanent Maturation arrest	Monocytopenia	X Linked	Xp11.4-p11.21	*WAS*	Cytoskeleton homeostasis
	
	WHIM [99]	193670	Severe permanent No maturation arrest Myelokathexis	Lymphopenia Thrombocytopenia	Dominant	2q21	*CXCR4*	Chemokine receptor (CXCL12)

Congenital neutropenia with extra hematopoietic manifestations	Kostmann' disease [3,4,53,232,233]	202700	Maturation arrest	Central nervous system: mental retardation/seizures	Recessive	1q21.3	*HAX1*	Anti-apoptotic protein located in mitochondria and in the cytosol
	
	Shwachman-Bodian-Diamond disease [65]	260400	Mild neutropenia Dysgranulopeosis mild dysmegacacyopoeisis	Exocrine Pancreas deficiency Bone: metaphyseal dysplasia Central nervous system: mental retardation Heart: cardiomyopathy	Recessive	7q11.22	*SDBS*	Ribosomal protein Regulation of RNA expression
	
	Severe congenital neutropenia [10]	202700	Maturation arrest	Skin -prominent superficial venous network Heart: atrial defect Uropathy	Recessive	17q21	*G6PC3*	Glucose 6 -phosphatase complex: Catalytic unit
	
	Barth disease [77]	302060	No maturation arrest	Hypertrophy cardiomyopathy	X Linked	Xq28	*TAZ (G4.5)*	Tafazzin: Phospholipid membrane homeostasis
	
	Hermansky- Pudlak syndrome type 2 [80]	608233	No maturation arrest	Albinism	Recessive	5q14.1	*AP3B1*	Cargo protein/ER traficking with *ELANE *interaction
	
	Neutropenia with AP14 mutation[78]		No maturation arrest	Albinism	Recessive	1q21	*AP14*	Lysosome packaging
	
	Poikilodermia type clericuzio[75,76]	604173	No maturation arrest Minor dysgranulopoetic features	Skin: poikilodermia	Recessive	16q13	*16ORF57*	Not known
	
	Glycogen storage type Ib [234]	232220	No maturation arrest	hypoglycemia, fasting hyperlactacidemia, and glycogen overload of the liver	Recessive	11q23.3	*SLC37A4*	Glucose 6 -phosphatase complex: Trans ER Transporter
	
	Cohen syndrome[74]	216550	No maturation arrest	psychomotor retardation, clumsiness, microcephaly, characteristic facial features, hypotonia and joint laxity, progressive retinochoroidal dystrophy, myopia	Recessive	8q22-q23	*VPS13B*	Sorting and transporting proteins in the ER

Diseases not usually assimilated to congenital neutropenia but including chronic neutropenia	IRAK 4 deficiency [95]	606883	Permanent mild but severe infection No maturation arrest	No	Recessive	12q12	*IRAK4*	Mediators of Toll-like receptor signal transduction
	
	Dominant Charot Marie Tooth disease[137,138]	602378	No maturation arrest	Axonal neuropathy type Charcot Marie Tooth Eyes: congenital cataract	Dominant	19p13.2-p12	*DNM2*	GTPases Regulation of the actin cytoskeleton
	
	Cartilage-hair hypoplasia [125]	250250	No maturation arrest	Dwarfism metaphyseal dysplasia Abnormal hair Lymphopenia aganglionic megacolon	Recessive	9p21-p12	*RMRP*	Endoribonuclease

**Table 2:** Main features and genetic subtypes of congenital neutropenia

System	Hematological or associated features	Disease	Gene
Blood/bone marrow maturation	Maturation arrest	*ELANE* *HAX 1* *WASP* *Neutropenia G6PC3* *GCSF receptor*	*ELANE* *HAX1* *WASP* *G6PC3* *Extra cellular domain of CSF3R*
	
	No maturation arrest	GSDIB WHIM Shwachman Diamond disease Cohen disease Hermansky Pudlak type 2	*G6PC T* *CXCR4* *SBDS* *VPS13B* *AP3B1*
	
	Myelokathexis	WHIM	*CXCR4*

Pancreas	External pancreatic insufficiency	Shwachman Diamond disease	*SBDS*

Eyes	Congenital cataract	Charcot Marie Tooth	*Dynamin 2*
	
	retinochoroidal dystrophy	Cohen disease	*VPS13B*

Heart	Heart: arrythmias	Neutropenia G6PC3	*G6PC3*
	
	Dilated Cardiomyopathy	Barth' diseases	*Tafazin*
	
	Cardiomyopathy	Shwachman Diamond disease	*SBDS*
	
	Various cardiac abnormalities	Shwachman Diamond disease WHIM Neutropenia G6PC3	*SBDS* *CXCR4* *G6PC3*

Skin	Skin xerosis eczema	Shwachman Diamond disease	*SBDS*
	
	Skin: prominent superficial veins	Neutropenia G6PC3	*G6PC3*

	Skin poikilodermia	SCN with poiikiloderma Type cleruzio	*16ORF57*
	
	Skin: Partial or complete albinism	Hermansky Pudlak type 2 AP14 defect Chediak Higashi disease Griscelli disease	*AP3B1* *AP14* *LYST* *RAB27A*
	
	Hair: fine, sparse and light-colored	Cartilage Hair hypoplasia	*RMRP*

Bone	Metaphyseal dysplasia	Shwachman Diamond disease Cartilage-hair hypoplasia	*SBDS* *RMRP*
	
	Facial Dysmorphia	Cohen disease	*VPS13B*

Central nervous system	Mental retardation	Kostmann's disease Shwachman Diamond disease Cohen disease	*Hax 1* *SBDS* *VPS13B*

Muscle	Weakness	Neutropenia G6PC3 Axonal Charcot Marie Tooth disease	*G6PC3* *Dynamin 2*

Metabolic pathway	Fasting intolerance and glycogenosis	Glycogen storage disease type Ib	*SLC37A4*

Inner ear	Inner ear defect	GFI 1/severe chronic neutropenia Reticular dysgenesia	*GFI1* *AK2*

Urogenital tract	Uropathy	Neutropenia G6PC3	*G6PC3*
	
	Cryptorchidism	Cohen disease Neutropenia G6PC3	*VPS13B* *G6PC3*

## Physiology of myeloid differentiation

Granulopoiesis is the physiological process by which circulating neutrophils are produced and regulated. Polymorphonuclear neutrophils or granulocytes (referred to below simply as 'neutrophils') are responsible, along with monocytic cells, for innate (naïve) immunity to bacteria and fungi, based on phagocytosis and the release of proteases, antimicrobial peptides and reactive oxygen species [37]. Neutrophils also play a role in inflammation and healing. This cellular system cannot be "educated", contrary to the lymphocytic system, and emerged early in phylogenesis, being identified in mollusks, for example, as early as 1891[38].

In vitro, antibacterial activity is tightly linked to the number of neutrophils, and is absent below a critical threshold [39].

The overall dynamics of the neutrophil system and tissular neutrophil distribution were investigated with radiolabeling methods in the 1960-1970s. These studies show that granulopoiesis takes between 7 and 13 days, and that neutrophils have a half-life, measured after ^32^P labeling, of about 5.4 to 6.7 hours in peripheral blood [40,41]. Circulating neutrophils represent only 3% to 5% of all neutrophils cells, and their total number is about 35 × 10^7 ^per kilogram. It is important to stress the highly dynamic nature of this system. In basal conditions, about 6 × 10^7^/neutrophils/kg are replaced every hour. Thus, circulating neutrophil analyses provide only a simple "snapshot" of the situation at a given moment. The soluble mediators (cytokines) that control this process started to be identified in the 1980s and late 1990s, along with their mechanisms of action and their interactions. These discoveries led to therapeutic development of G-CSF (Granulocyte Colony-Stimulating Factor) [42], which has vastly improved the management of patients with malignancies and hematologic disorders, including congenital neutropenia.

## Congenital neutropenia - classification and etiology

There is no simple consensus classification of congenital neutropenia. The genotype is the most important information for distinguishing one form of neutropenia from another, but it is not available during the initial work-up. The phenotype represents a continuum, with overlapping clinical manifestations: some important forms of organ involvement may not be present on initial examination. Table 1 shows associated disorders and likely diagnoses, while Table 2 lists the main diagnoses and affected organ systems.

### Neutropenia with no extra-hematopoietic manifestations and with normal adaptive immunity

#### *ELANE *(ELA2): Permanent and cyclic neutropenia

*ELANE *(neutrophil Elastase) mutations are the most frequent known cause of congenital neutropenia and are observed in two subtypes: congenital or permanent severe neutropenia, and cyclic neutropenia. They are found in about 40% to 55% of patients with congenital neutropenia [43,44].

Permanent neutropenia, usually called severe congenital neutropenia, is associated with deep-seated bacterial and fungal infections, stomatologic disorders, neutropenia usually below 0.2 G/l, monocytosis, hypereosinophilia and hypergammaglobulinemia, and sometimes with inflammatory anemia and maturation arrest of granulocytic cells at the promyelocyte stage (Additional file 1, Figure S1 Plate #3). These patients require large doses of G-CSF, both for the management of active infections and as long-term therapy. There is a high risk of leukemic transformation in this setting. Severe congenital neutropenia is usually diagnosed before age 6 months.

Cyclic neutropenia is less severe. The diagnosis is generally raised during the second year of life, or later, and the main clinical manifestation is recurrent acute stomatologic disorders (especially aphthae). The bone marrow aspect is variable over time (especially the granulocytic cell maturation pyramid), and is sometimes strictly normal.

Cyclic neutropenia nevertheless carries a risk of serious infections: the cumulative risk of experiencing at least one serious (potentially life-threatening) infection by age 20 years is similar in patients with permanent and cyclic neutropenia, although the former patients tend to have earlier manifestations.

No recurrent extra-hematopoietic disorders have been described in *ELANE *neutropenia.

By comparison with other forms of congenital neutropenia, neutropenia due to *ELANE *mutations is associated with the most severe infectious complications [43].

As the same mutations can be responsible for both types [43], and taking into account serial blood cell counts in patients with apparently cyclic or permanent neutropenia, the two subtypes can be considered as part of a continuum of the same disease. In addition, a given family may include members with very severe permanent neutropenia or more cyclic forms.

*ELANE *mutations were identified in 1999 by linkage analysis and positional cloning in 13 families with a long history of cyclic neutropenia with autosomal dominant transmission [8]. *ELANE *is a serine protease that cleaves elastin, among other proteins and its physiological inhibitor is α1-antitrypsin. *ELANE *is homologous to two other proteases produced by polymorphonuclear cells: proteinase 3 (the target of anti-neutrophil cytoplasm antibodies present in Wegener's disease) and azurocidin [45]. These three proteins, whose genes lie next to one another in chromosome region 19p13.3, are jointly regulated. *ELANE *is selectively stored in neutrophil azurophil granules, starting at the promyelocyte stage, but may also be found at the cell surface or within the cytoplasm.

Soon after the discovery of their involvement in cyclic neutropenia, *ELANE *mutations (about 50 listed to date) were also identified in patients with severe congenital neutropenia [9].

Some mutations creating a premature stop codon and leading to the synthesis of a truncated protein (lacking the last exon) are observed only in severe permanent congenital neutropenia. The G185R mutation is responsible for very severe phenotypes [43,46].

The effects of these mutations on the protein are poorly documented. Mice with no *ELANE *gene expression or carrying mutations associated with severe congenital neutropenia in humans are not neutropenic [47]. Similarly, no correlation has been found between specific mutations and the protein's enzyme activity. In contrast, abnormal protein folding and cytoplasmic protein accumulation have been described [47-51]. Our understanding of the impact of *ELANE *mutations on intracellular protein trafficking, and particularly on granule packaging, has benefited from investigations of a genetic disease with very similar features and involving the gene coding for AP3 protein. This "cargo" protein is responsible for intraluminal trafficking of proteins from the Golgi apparatus to lysosomes, including neutrophil granules. Mutations of the AP3 tetramer subunit in humans are responsible for the Hermansky-Pudlak syndrome type 2, associated with partial albinism, and for cyclic neutropenia in Grey Collie dogs, considered the best animal model of cyclic neutropenia. *ELANE *mutations inhibit AP3 protein binding, thereby hindering its packaging [49]. This phenomenon contributes to endoplasmic reticulum stress through the unfolded protein response [48,51]

#### Extracellular G-CSF receptor defects

No more than 5 cases have been reported to date. Here the clinical picture [52] is very similar to that of severe congenital neutropenia due to *ELANE *mutations, but this disorder is entirely unresponsive to G-CSF, even at doses up to 100 μg/kg per day. No constitutional anomaly common to all cells has so far been identified and this entity can be considered as a somatic mutant.

### Congenital neutropenia with extra-hematopoietic manifestations

#### Kostmann's syndrome and *HAX1 *mutations

The disorder, described by Rolf Kostmann in 1950 and 1956 [1,2], remains a paradigm in the field of congenital neutropenia. The term Kostmann's syndrome is sometimes used, inappropriately, for neutropenia with *ELANE *mutations.

The exact frequency of this entity is not precisely known but appears to be far lower than *ELANE *neutropenia, except in some geographic areas such as Sweden and Kurdistan.

The main clinical features are severe neutropenia with monocytosis and reactive eosinophilia and strong susceptibility to bacterial infections (11 deaths occurred before age 1 year among the 14 patients initially described). The pedigree lived in an isolated geographic area (northern Sweden) and involved consanguineous families, pointing to monogenic autosomal recessive transmission. A later publication by Kostmann, in 1975 [33], focusing on the same pedigree, showed improved survival thanks to the use of antibiotics, but also the onset of neurological disorders in the second decade, with both mental retardation and seizures. This syndrome is better described in a more recent study of the same pedigree, in which 5 of the 6 patients had neurological disorders [53]. Neurological involvement may depend on the mutation [54].

The molecular bases of this entity were discovered by classical genetic linkage analysis of three Kurdish families (two of which were consanguineous), followed by fine mapping of the region of interest on chromosome arm 1q, leading to the identification of *HAX1 *(HS1-associated protein X1) as the gene responsible for the disease. The mutations were different in the Kurdish families and the patients from the family described by R. Kostmann in 1956. *HAX1 *(35 kDa) is a ubiquitous mitochondrial protein with multiple partners. It has anti-apoptotic properties, due to mitochondrial membrane potential stabilization. These patients' neutrophils, and also their fibroblasts, are very sensitive to apoptotic stimuli, and this anomaly can be corrected in vitro by restoring a normal *HAX1 *protein level in CD34+ bone marrow myeloid progenitors, and in vivo through the anti-apoptotic function of G-CSF.

#### Shwachman-Diamond syndrome

This is quite a frequent form of congenital neutropenia, representing one-quarter of all cases of congenital neutropenia recorded in the French Congenital Neutropenia Registry.

First described by Nezelof in 1961 [55] and then by Shwachman and Diamond in 1964 [56], Shwachman-Diamond syndrome associates hematologic disorders with a malformative syndrome, the most consistent feature of which is external pancreatic insufficiency due to fatty involution, yielding a characteristic pancreatic aspect on magnetic resonance imaging [57], as well as chronic diarrhea with fat stools and low fecal elastase. Other features include cutaneous involvement (usually eczema, but sometimes icthyosis), bone involvement with metaphyseal dysplasia and narrow thorax [58], and psychomotor retardation [59]. Neutropenia is usually intermittent and moderate, with a decline in chemotactism associated with mild to moderate thrombocytopenia, moderate anemia, and a rise in fetal hemoglobin. The hematologic disorders can be complicated by bone marrow aplasia or leukemic transformation, mainly consisting of acute myeloid leukemia (FAB type 5 or 6), or a myelodysplastic syndrome with cytologic abnormalities (usually clonal), frequently affecting chromosome 7 (Additional file 1, Figure S1 Plates #4, #5, #6) [21,60].

The predominant clinical manifestations are highly variable. Neonatal forms have been described, with respiratory distress, narrow thorax, pancytopenia [61,62], and especially neurological involvement (mental retardation) [63], predominant gastrointestinal disorders (gluten intolerance), growth retardation in the second year of life, and predominant bone involvement suggestive of a constitutional bone disorder [64]. Depending on the presenting manifestations, differential diagnoses include Cystic fibrosis, Pearson's syndrome (characterized by cytologic abnormalities and especially mitochondrial respiratory chain defects), Fanconi anemia (distinguished by the constitutional karyotype) and gluten intolerance.

The genetic defect underlying the Shwachman Diamond syndrome has now been identified [65]. It involves the *SDBS *gene located on chromosome 7. This ubiquitously expressed gene encodes a ribosomal protein involved in the traduction process [66]. Nearly 98% of patients with this syndrome have mutations of the *SBDS *gene. Despite marked clinical polymorphism, the mutations are limited in number (practically always double heterozygous mutations) and the p.Lys62X/p.Cys84fs mutation is present in two-thirds of patients.

#### Glucose-6-phosphatase complex disorders: glycogen storage disease type Ib and G6PC3

Genetic studies show that the two entities are closely related, despite very different clinical phenotypes. Both feature neutropenia. Glycogen is stored in the liver and, after glycogenolysis, can yield glucose-6-phosphate, which can be used directly for energy production (glycolysis) or be dephosphorylated (by glucose 6 phosphatase) to yield glucose, which can be transported throughout the body to meet cellular energy needs.

Glucose 6 phosphatase is a complex of three proteins bound to the endoplasmic reticulum. Two of these three proteins are involved in congenital neutropenia: the translocase (*SLC37A4*), previously named G6PT1, transports glucose 6 phosphate between the cytoplasm and the lumen of the endoplasmic reticulum, while G6PC3 is a catalytic protein.

The most remarkable feature of the association between these molecular abnormalities and neutropenia is the fact that the glycogenolysis pathway and, more generally, the glucose 6 phosphatase metabolic pathway, is not the usual energy source in neutrophils, which mainly use the pentose pathway.

##### Neutropenia associated with glycogen storage disease Ib

Glycogen storage disease type Ib is characterized by metabolic disorders common to all forms of glycogen storage disease type I (hepatic glycogen accumulation, intolerance of fasting, hypoglycemic events, and hyperlactacidemia), as well as susceptibility to infections [67], and colitis resembling Crohn's disease both clinically and radiologically [36].

This susceptibility to infections is due to neutropenia and, sometimes, to neutrophil dysfunction (mainly defective chemotactism). Bone marrow smears show hyperplasia of the granulocytic lineage, without maturation arrest (Additional file 1, Figure S1 Plate #7). The origin of the neutropenia and neutrophil dysfunction is not known. It is not related to nutritional status and is not corrected by liver transplantation [68]. This, and the lack of any known role of the Gluco 6 Phosphate translocase (gene *SLC37A4*, previously named G6PT1), in neutrophil energy metabolism, raises the possibility that this protein has another function in neutrophils. Gene therapy in a mouse model has corrected both the metabolic and myeloid disorders [69].

##### Neutropenia associated with *G6PC3 *mutations

This entity associates severe permanent neutropenia with granulocyte maturation arrest, susceptibility to infections, and several other clinical manifestations, including thin skin with a highly visible veins, urogenital malformations, and cardiac disorders (especially arrhythmia due to defective atrioseptal conduction); some patients have a myopathic syndrome (despite a normal histologic and microscopic aspect of muscle) or perception deafness. Mutations of the *G6PC3 *gene are generally homozygous, but a double heterozygote has been described [10] and corresponds to an animal model [70]. Homozygous mutations have been shown to affect the endoplasmic reticulum [71].

#### Cohen's syndrome

A very rare form of congenital neutropenia, this autosomal recessive syndrome associates mental retardation with a dysmorphic syndrome that includes microcephaly, facial abnormalities (moon face), myopia, pigmentary retinitis, trunk obesity, and ligament hyperlaxity [72]. Neutropenia is present in over 90% of cases and is responsible for chronic infections with gingivostomatitis. The marrow is rich, with no maturation arrest [73]. Cohen's syndrome has been linked to mutations of the *VPS13B *gene, located on chromosome 8 and coding for an endoplasmic reticulum protein [74].

#### Neutropenia associated with poikilodermia, Clericuzio type

The poikilodermia includes skin atrophy and a papular erythematous rash. Several subtypes of this genodermatosis have been described.

The Clericuzio type was first described in Navajo Indians. Onset occurs in the first year of life. The rash gradually propagates centripetally from the limbs and comprises a papular rash, followed by plaques of hypo- and hyperpigmentation and telangiectasies. The nails are affected too (pachyonychia), but no hair loss or leukoplasia is observed. Recurrent infections occur, and especially pneumonia.

The neutropenia is often severe. Granulocyte maturation arrest at the promyelocyte stage is rarely observed, but dysgranulopoiesis with late arrest is often seen [75]. An Italian linkage study [76] revealed composite mutations of the *C16ORF57 *gene, whose function is unknown.

#### Barth's disease

This X-linked syndrome combines dilated cardiomyopathy with endomyocardial fibrosis (sometimes leading to early death), myopathy and moderate or profound neutropenia, sometimes responsible for severe infections. There is also an acidopathy involving several organic acids, including 3-methylglutaconic acid. This condition is due to mutations in the *G4-5 *gene, which encodes *tafazzin*, a protein involved in phospholipid membrane homeostasis [77].

#### Neutropenia and albinism: AP14 deficiency

Several children of a consanguineous Mennomite family presented with partial albinism, severe neutropenia and susceptibility to pneumococcal infection. Bone marrow studies showed no maturation arrest and there were no shared morphological features with Griscelli or Chediak Higashi disease. This syndrome, which has so far been detected in only one family, is due to deficiency of a protein (AP14) involved in intracellular endosome trafficking [78].

#### Neutropenia and albinism: Hermansky Pudlak syndrome type 2

Hermansky Pudlak syndrome was first described in 1959, in patients with partial albinism, hemorrhagic complications and platelet granulations. In 1994, a similar syndrome associated with neutropenia was described [79]. This entity, known as Hermansky Pudlak syndrome type 2, is due to mutations in the *AP3 *cargo protein [80]. It is the canine equivalent of Grey Collie cyclic neutropenia [81]. To understand the packaging function of *AP3*, it was first necessary to elucidate the effects of neutrophil elastase mutations, as the two proteins interact during granule packaging [49,82].

#### Miscellaneous malformative syndromes

Several distinct phenotypic entities combine neutropenia and a variety of other conditions, including trichothiodystrophy [83], cuti laxa, uropathy, cardiopathy [84], and Klippel Trenaumay syndrome [21]. No noteworthy genetic mutations have been found in these isolated cases.

### Chronic neutropenia with defective naive/adaptive immunity, considered as congenital neutropenia

Multiple interactions take place between the innate and adaptive immune systems. Toll receptors are shared by the two systems, while some proteins expressed by the phagocyte lineage are involved in the lymphocyte lineage [85]. Some metabolic pathways and multiple effectors (e.g. interleukins) are also shared. This explains why many "lymphocyte disorders" can also be associated with neutropenia. Indeed, these associations are so frequent [86] that both adaptive immunity and other functions of the innate immune system must be investigated when chronic neutropenia is diagnosed. These morbid associations, often attributed to viral infections or autoimmunity, also involve common pathophysiologic mechanisms, as shown by studies of some extremely rare disorders like Bruton's disease [87].

#### Neutropenia associated with GFI1 mutations

This is an extremely rare cause of congenital neutropenia, so far described in only four patients [44,88]. The clinical phenotype does not seem to be very homogeneous, as one patient was diagnosed with severe neutropenia at 4 months of age, together with marked monocytosis, while the father, who had the same mutation, had moderate, asymptomatic neutropenia, and the second patient, diagnosed at age 56 years with idiopathic neutropenia, had no clear susceptibility to infections. These patients all have moderate lymphopenia (CD3 cells between 1 and 1.4 G/l) with normal memory cells and humoral immunity.

GFI1, a nuclear protein, is a transcriptional repressive factor involved in T lymphomatogenesis and in the development of T cell progenitors. Its involvement in granulopoiesis and in macrophage activity has been demonstrated in knock-out mice, which also exhibit an inner-ear defect. Heterozygous *GFI1 *mutations, which are dominant mutations, lead to an increase in *ELANE *expression, in the same way as *ELANE *mutations.

#### Permanent congenital neutropenia due to Wiskott-Aldrich syndrome (WAS) gene mutation

This is also a very rare entity observed to date in 5 families. Its hematologic and infectious features resemble those of *ELANE *neutropenia, but with no monocytosis despite profound neutropenia [89-93]. Some cases are only diagnosed in adulthood, implying that some patients have limited infectious complications. This is an X-linked disorder. A genetic linkage study of a pedigree with suspected sex-linked genetic transmission revealed mutations in the *WAS *gene (encoding Wiskott-Aldrich syndrome protein) in a family with severe congenital neutropenia [92], and more recently in four other families [44,89-91,94].

These patients' phenotype is completely distinct from that of patients with the classical form of Wiskott-Aldrich syndrome, which comprises eczema, thrombocytopenia with small platelets, and immune deficiency.

This phenotypic difference, despite the shared involvement of the *WAS *gene, is due to functional differences in the respective mutations (*WAS *protein activation in congenital neutropenia and defective *WAS *protein activity in the classical syndrome).

As *WAS *protein is involved in intracytoplasmic actin polymerization, mutations observed in patients with neutropenia lead to an increase in actin polymerization, accompanied by an increase in the podosome level and in apoptosis.

#### Neutropenia associated with *IRAK 4 *mutations

A deficiency in interleukin 1 receptor-associated kinase 4 leads to a functional defect of innate immunity [95]. It includes marked susceptibility to pyogenic infections (especially staphylococci and pneumococci), but no other extra-hematologic or infectious manifestations. These patients have only moderate neutropenia, which tends to normalize during infections. However, functional tests, and especially the monocyte response to various stimuli, such as LPS, show defective neutrophil and monocyte mobilization [96], whereas standard immunological findings can be normal.

#### NK cell deficiency and neutropenia

NK cell deficiency and dysfunction have been described in four patients with chronic neutropenia and maturation arrest at the promyelocyte stage in the only relevant study. These findings were made before the main molecular abnormalities were identified [97]. It is impossible to know whether this feature is common to several forms of congenital neutropenia or whether it represents an original entity.

#### Wart hypogammaglobulinemia immunodeficiency myelokathexis (WHIM) syndrome

This form of constitutional neutropenia is characterized by morphological abnormalities of the rare circulating neutrophils, which are hypersegmented and contain cytoplasmic vacuoles; bone marrow cells show similar anomalies (Additional file 1, Figure S1 Plate #8). This unusual morphological aspect (kathexia meaning neutrophil retention in the bone marrow) justified the use of the initial term [98]. Later, immunological abnormalities were also reported, including lymphopenia and moderate hypogammaglobulinemia [99]. Severe papillomavirus warts are almost always present, leading to the adoption of the term "wart hypogammaglobulinemia immunodeficiency myelokathexis". Subsequent identification of the role of a chemokine receptor gene (*CXCR4*)[100] led to a better understanding of this disease and showed that this syndrome corresponds to the same entity, although warts may not initially be present. CXCR4 is a chemokine receptor known for its role as an HIV coreceptor [101]. This receptor and its ligand SDF1 (CXCL12) are involved in organogenesis, B lymphocyte ontogenesis, and myelopoiesis, and are required for CD34+ cell migration from bone marrow. Mutations of the *CXCR4 *chemokine are dominant mutations, leading to receptor hyperactivity and defective mobilization of bone marrow neutrophils (myelokathexis) and lymphocytes.

### Neutropenia associated with miscellaneous constitutional disorders NOT considered as congenital neutropenia

Neutropenia is not a major clinical or biological feature of these disorders. They are not usually considered to be forms of congenital neutropenia, because the neutropenia is transient (for example in Bruton's agammaglobulinemia), or tends to occur late, or is only moderate and does not require any particular management (for example Charcot and Tooth disease with *dynamin 2 *mutation).

#### Chronic neutropenia, with defective innate/adaptive immunity NOT considered as congenital neutropenia

##### Humoral immune deficiencies

Bruton's agammaglobulinemia (~30% of cases), CD40 ligand deficiency (immune deficiency with IgM hypergammaglobulinemia, 50% of cases), variable hypogammaglobulinemia and unclassified hypogammaglobulinemia can be accompanied by neuropenia [86,97,102-105]. The neutropenia is usually detected before immunoglobulin replacement therapy and responds to immunoglobulin therapy [106]. In Bruton's agammaglobulinemia, due to *BTK *gene mutations, the neutropenia can be very profound at onset, with maturation arrest at the promyelocyte stage. Humoral immunity should be thoroughly investigated in patients with neutropenia.

##### Severe combined immune deficiency and immune deficiency syndromes

Severe combined immune deficiencies (like those associated with IL-2 receptor gamma mutation) can also include neutropenia. The lymphocyte deficit, mainly affecting T cells [107], frequently includes neutropenia, which can be severe. Other immune deficiencies that are not as severe at onset, such as defective HLA class II expression and ataxia-telangiectasia, can also include neutropenia. In Wiskott-Aldrich disease, neutropenia usually accompanies the frequent autoimmune disorders [108], through a mechanism different from that underlying X-linked neutropenia and activating *WASP *mutations.

##### Reticular dysgenesis and *AK2 *gene mutation

Reticular dysgenesis is an autosomal recessive form of the severe combined immune deficiency syndrome (SCID) affecting hematopoietic lineages of both the innate and adaptive immune systems. At birth, this condition is characterized by a total absence of neutrophils, T cells and NK cells, sometimes associated with anemia, thrombocytopenia and low B cell counts, while monocyte counts remain normal. This disorder also affects the inner ear, leading to profound perception deafness. Recently, the gene responsible for this form of SCID was identified by two independent teams [109,110]. It codes for adenylate kinase 2 (*AK2*), a ubiquitous enzyme involved in energy metabolism and whose known function is reverse transphosphorylation of AMP and ATP into ADP.

##### 22q11 syndrome

This is a complex malformative syndrome due to interstitial deletion of chromosome 22 at the q11 locus. Few children present all the characteristic features of this syndrome simultaneously. ENT disorders comprise velar insufficiency, facial malformation (especially of the lower face), sometimes accompanied by marked retrognatism. Other disorders include parathyroid deficiency with hypocalcemia, cardiac abnormalities (especially tetralogy of Fallot) and immunologic abnormalities, including, in the most severe cases, Di George syndrome with thymic agenesis, and T lymphocyte deficiency. Platelet disorders have been described [111,112] and also neutropenia, sometimes of an autoimmune nature [113].

##### Exocytosis disorders

Neutropenia is found in several cellular exocytosis disorders [114], leading to hemophagocytic lymphohistiocytosis (HLH) but also sometimes inaugural neutropenia, as in *AP14 *mutation disorders and Hermansky Pudllak disease type 2. Most genetic defects associated with these disorders have now been identified, and we will only recall the main phenotypes, of which the principal extra-hematopoeitic manifestation is complete or partial albinism [115-117].

##### Chediak Higashi syndrome (CHS)

CHS is characterized by partial oculo-cutaneous albinism, abnormal melanosome hair repartition, giant granules in all neutrophils and in bone marrow granulocytic precursors (Additional file 1, Figure S1 Plate #9), bright red inclusions in some lymphocytes, defective bactericidal activity, and NK dysfunction. Neutropenia due to bone marrow destruction occurs early in this disorder, prior to HLH.

##### Griscelli syndrome type 2 (GS2)

The clinical manifestations of this disorder share many features of Chediak-Higashi syndrome, especially albinism and immune deficiency, and sometimes HLH in GS2, and not in GS1 and GS3 (Additional file 1, Figure S1 Plate #10). It differs by the absence of giant granulations in blood cells, and the microscopic aspect of the hair. Neutropenia can be present, either in isolation or during the course of a macrophage activation syndrome.

##### Familial hemophagocytic lymphohistiocytosis (FHLH)

These inherited immune dysregulation syndromes are related to mutations in *perforin*, *Munc13.4*, *Munc18.2 *or *Syntaxin11 *encoding genes and are defined by early onset of severe HLH. Neutropenia is one of the diagnostic criteria of HLH, though not a major feature. Morphological anomalies are rare.

#### Other syndromes associated with neutropenia

##### Blackfan-Diamond anemia

Several years after onset, neutropenia can occur in patients with Blackfan-Diamond anemia.

##### Fanconi anemia and dyskeratosis congenita

Neutropenia is an integral feature of these constitutional forms of bone marrow aplasia, which are associated with complex malformations. Anemia and thrombocytopenia, but rarely neutropenia, are present at diagnosis.

##### Constitutional monosomy 7

Constitutional monosomy 7 has been found in several patients with sporadic or familial neutropenia. Secondary malignant transformation is the rule [118-120].

##### Aminoacidopathies

Neutropenia is a secondary feature of several aminoacidopathies, including hyperglycinemia, and isovaleric, propionic and methylmalonic acidemia [121]. Chronic fluctuating neutropenia is a feature of dibasic protein intolerance (also called lysinuric protein intolerance) and there is a typical cytologic aspect (Additional file 1, Figure S1 Plate #11). Other features of the macrophage activation syndrome are also present [122].

##### Pearson's syndrome

Pearson's syndrome associates external pancreatic insufficiency with pancytopenia. Neutropenia can be present, together with anemia and thrombocytopenia. This syndrome is due to a mitochondrial respiratory chain disorder and to mitochondrial DNA deletion [123]. The neutropenia is usually less severe than the anemia. The diagnosis is raised by sideroblastic anemia, with evocative cytologic abnormalities (Additional file 1, Figure S1 Plate #11) and unexplained acidosis.

##### Cartilage-hair hypoplasia

This syndrome combines dwarfism, metaphysal chondrodysplasia, sparse hair, and sometimes an immune deficiency, with lymphopenia, hypogammaglobulinemia and neutropenia [124]. This autosomal recessive disease, mainly affecting the Amish (USA) and Finnish populations, is due to mutations of the *RMRP *gene, coding for a ribonuclease [125].

##### Chronic neutropenia, recurrent fever, Behçet's disease and amyloidosis

Recurrent fevers are a set of disorders comprising recurrent fever, various inflammatory manifestations (serous and articular) and sometimes recurrent aphthosis.

Amyloidosis is a common complication of these disorders, and especially of familial mediterranean fever (FMF) [126]. Hyperleukocytososis is usually present [127], but an authentic case of FMF with neutropenia has been described [128].

Congenital neutropenia is often associated with hypergammaglobulinemia and a chronic inflammatory syndrome, but secondary amylosis (AA type) is very rare [129-131]. The particularities of these patients suggest that this is an independent entity.

Behçet's disease is distinct from recurrent fever, but the two disorders share the same geographic predominance (Mediterranean basin) and certain traits such as recurrent aphthae, as in neutropenic disorders. Polynucleosis is common, but cases with associated neutropenia have been reported [132].

##### Finnish nephrotic syndrome

The "Finnish" nephrotic syndrome is an autosomal recessive disorder defined by structural modification of nephrin, leading to massive renal protein leakage. An extremely severe nephrotic syndrome (albuminemia < 10 g/l) and massive proteinuria are present from birth.

Neutropenia can also occur in this setting [133,134]. It is due to leakage of proteins, and especially ceruloplasmin (the protein responsible for copper transport), leading to very low circulating copper levels.

As shown in an animal model [135], copper deficiency can lead to severe neutropenia, with maturation arrest of granulopoeisis at the promyelocyte stage, as in typical congenital neutropenia. Copper administration suffices to correct the deficiency and to restore a normal neutrophil count [136].

##### Charcot-Marie-Tooth disease and *dynamin 2 *mutations

Charcot-Marie-Tooth disease comprises a variety of neurological disorders with hereditary sensory-motor neuropathy. Life expectancy is unaffected and there is no mental retardation.

Schematically, CMT is due to damage to the peripheral nerves connecting the spinal cord to the muscles, affecting nerve conduction. This leads to gait disorders, cramps and frequent foot deformation. CMT can occur during childhood but sometimes also in adulthood. In general, CMT deteriorates slowly, but it can also progress by exacerbations. There are several types, currently classified according to the affected part of the nerve (myelin or axon) and the mode of transmission (dominant or recessive). Type II is characterized by axonal involvement. In this form, with dominant transmission related to the mutation in the *dynamin 2 *gene, neurological signs are sometimes discreet and are accompanied by congenital cataract and fluctuating neutropenia; the neutropenia is usually mildly symptomatic but it may be severe and is sometimes the initial manifestation [137,138].

## Diagnosis of congenital neutropenia

Neutropenia is a relatively frequent finding, while congenital neutropenia is quite rare. Neutropenia is often well tolerated and normalizes rapidly, in which case specialized investigations are not necessary. Neutropenia is sometimes a secondary finding in a patient with far more significant disorders, who may thus be at risk of infectious complications. More rarely, neutropenia persists and/or emerges as the sole cause of a child's symptoms, in which case thorough investigations are necessary.

The interview and physical examination may reveal a particular etiology, such as a viral infection or malignant hemopathy, an iatrogenic cause, or an immune deficiency, warranting further specific investigations.

In non urgent settings, the permanent, intermittent or regressive nature of the neutropenia should be established during an observation period of a few weeks, in which the number of infections and any changes in buccal disorders (ulceration, gingivitis, etc.) should be noted, as they can help guide patient management.

Bone marrow examination is often necessary to rule out malignant hemopathies, determine cellularity, assess myeloid maturation, and detect signs of a precise etiology. Figure 1 shows a) the normal pyramid of granulocyte precursor maturation and b) early arrest at the promyelocytic stage (in a patient with *ELANE *mutation). Maturation arrest at the promyelocyte stage is often associated with bone marrow hypereosinophilia and monocytosis. Morphologically, few aspects are truly typical of a particular etiology. Specific hemophagocytosis of neutrophils is a sign of autoimmune neutropenia in young children (Additional file 1, Figure S1 Plate #13)[139-141], while cytoplasmic granulations are suggestive of Chediak Higashi disease (Additional file 1, Figure S1 Plate #9), hemophagocytosis points to dibasic protein intolerance(Additional file 1, Figure S1 Plate #11) and myelokathexis, defined by an increase in the granulocyte pool with hypermature and dystrophic features (Additional file 1, Figure S1 Plate #8) point to WHIM syndrome. Finally, precursor vacuolization is a sign of Pearson's syndrome (Additional file 1, Figure S1 Plate #12). The marrow smear may reveal non specific dysgranulopoeisis or be totally normal, but this does not rule out a diagnosis of congenital neutropenia. Cytogenetic bone marrow studies are now crucial when investigating isolated neutropenia that is suspected of being congenital.

Several other investigations are of interest, especially antineutrophil antibody assay, immunoglobulin assay (Ig GAM), lymphocyte immunophenotyping, pancreatic markers (serum trypsinogen and fecal elastase) and liposoluble vitamin levels (vitamins A, E and D).

The glucagon challenge test and studies of neutrophil demargination are rarely used now, as they are complex and provide little information of practical use.

**Figure 2:**
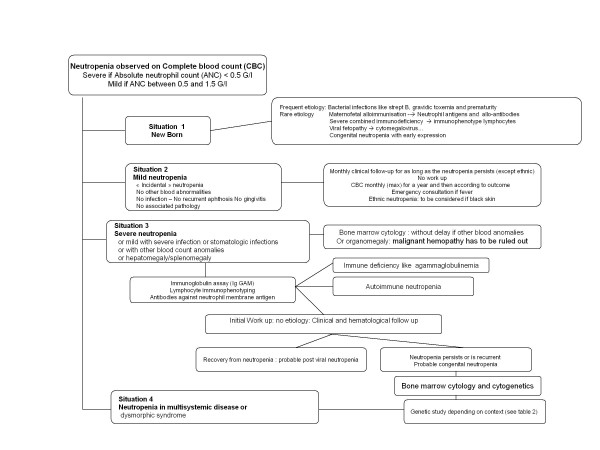
**Neutropenia: diagnostic tree**.

The recommended diagnostic work-up for neutropenia is shown in Figure 2, while table 3 lists the main forms of acquired neutropenia.

**Table 3:** Acquired neutropenia - main causes and method of the diagnosis

Main category	How to made the diagnosis	Causes in detail (not exhaustive...)
**Drug related neutropenia**	questioning Safety data	Cytostatic drugs - almost all except asparaginase **Anti-infectives **penicillins cephalopsorins sulfamids zidovudine acyclovir lévamisole pyrimethamine **tranquilisants **chlorpromazine phenothiazines **anti seizure **phenytoin arbamazepine **antithyroid drugs **propylthiouracil **Cardio vascular drug **procainamide quinidine **Anti rheumatic drugs Gold salts Non steroid ant inflammatory drug **colchicine aminopyrine D penicillamine

**Toxic**	Context/questioning	Benzene Ionizing radiation

**Infection**	Germ isolation or serology or any other probes	Typhoid Brucellae gram negative septicemia Mycobacterium Tuberculosis HIV EBV CMV Parvovirus varicela/Zoster A B C hepatitis,. almost all virus Leishmaniasis paludism

**Acquired malignant or benign hemopathy**	Bone marrow smear Bone marrow trephine Bone marrow cytogenetic	**Acute leukemia** **Bone marrow metastases** **Aplastic idiopathic anemia** **Myelodysplasia** **Macrophage activation syndrome/hemophagocytic lymphohistiocytosis**

**auto-immunity**	Auto Antibodies/Bone marrow smear - almost normal but sometimes Neutrophil Hemophagocytosis	Primitive or secondary to rhumatoid disease like in Felty syndrome

**Large granular lymphocytosis**	Blood cytology (> 0.4 G/l LGL) Immunophenotype Lymphoid clonality	**Large Lymphocyte Hyperlymphocytosis**

**Endocrinopathy**	Hormonal dosage	Hyper/Hypothyroidy Surrénal deficiency Pan hypopitutarism

**Nutrition deficiency**	Clinical examination Body mass index Vitamin and oligo element dosage	Anorexia nervosia, Marasmus, Copper insufisiency..

**Idiopathic**	No others cause	

## Differential diagnosis and some frequent causes of chronic neutropenia

### Allo-immune neutropenia

This form of neutropenia is present from birth and can be considered congenital.

It may be suspected following a maternofetal infection or a routine blood cell count. Initially severe (<0.1 G/l), it usually normalizes after 3-6 months. Available bone marrow studies show no maturation arrest. Neonatal allo-immune neutropenia is due to fetomaternal incompatibility for a paternally derived neutrophil antigen. By crossing the placental, these fetal neutrophils can elicit IgG antibodies in the mother, which then enter the fetal circulation. Based on fine characterization of neutrophil antigens, a new nomenclature (HNA, Human Neutrophil Antigens) has been proposed, with 5 systems. The first system, HNA1, codes for RFcγIIIb (CD16), a low-affinity IgG receptor, is polymorphous and includes three antigens: HNA1a, 1b and 1c. The genetic CD16 deficit on neutrophils and in its soluble form can lead to iso-immunization of the mother and neutropenia in the newborn. The second system, HNA-2, includes only one serologically defined antigen, HNA-2A, present on neutrophil subpopulations. The alloantibodies most frequently responsible for neonatal neutropenia are directed against the antigens of the HNA-1 system and, to a lesser extent, HNA-2. In rare cases, neonatal immune neutropenia can be due to a maternal IgG autoantibody. Diagnosis is based on the identification of a maternal antibody reacting selectively with neutrophils belonging to the panel expressing the antigen and\or with paternal neutrophils.

### Autoimmune primitive neutropenia

This is the most frequent cause of chronic neutropenia in children, and is better known under the term "benign chronic neutropenia" [142-144]. This form of isolated neutropenia is usually discovered after a moderately severe infectious episode in a small child (median age 8 months). Monocytosis, eosinophilia and/or moderate splenomegaly can be found. This neutropenia is permanent, at least for several months, ordinarily very profound, but it is usually well tolerated. The marrow smear shows hyperplasia of the granulocyte lineage, sometimes with late arrest. Macrophagia of intramedullary polymorphonuclear cells is a diagnostic sign [139-141]. The detection of anti-polymorphonuclear cell antibodies necessitates repeated examinations (only about 75% of cases are positive on the first examination). Several techniques can be used (detection of circulating antibodies or antibodies adherent to polymorphonuclear cells). The autoimmune process targets the same membrane glycoproteins on polymorphonuclear cells as those involved in autoimmune neutropenia. The most frequently involved is the receptor for the gammaglobulin invariable fragment (FcRgIIIb) or CD16, that is encoded by two co-dominant alleles (HNA-1A and HNA-1B, formerly NA 1 and NA 2). The infectious consequences are limited, probably because bone marrow reserves are unaffected by the autoimmune process. The neutropenia resolves spontaneously after 12 to 24 months (36 months in a few cases). It is rarely associated with another autoimmune disease or with an immune deficiency. It can be secondary to a viral infection. The adult form differs from the childhood form by its greater severity. Cytologic studies sometimes show early maturation arrest of the granulocyte lineage. In particular, the frontiers between autoimmune neutropenia, idiopathic neutropenia, and neutropenia associated with proliferation of large granular lymphocytes (LGL) are still rather vague in adults [145-147].

### Secondary autoimmune neutropenia

This form is rare in children, contrary to adults. The causes are numerous, but immune deficiencies are at the forefront. Neutropenia is generally a secondary feature, as for example in acute disseminated lupus erythematosus, and rheumatoid arthritis (especially Felty syndrome) [146,147]. Finally, autoimmune neutropenia associated with autoimmune involvement of another blood lineage corresponds to the definition of Evans syndrome [148].

### Idiopathic neutropenia

This diagnosis is generally made in adulthood [146]. Etiologic investigations are negative. The presence of anti-polymorphonuclear cell autoantibodies must be eliminated by repeated testing at intervals of several weeks, along with rare causes such as the association with a thymoma [149]. It seems that some of these neutropenias are associated restriction of T lymphocyte clonality, thus resembling hyperlymphocytosis with large granular lymphocytes [150]. Several pediatric cases of large granular lymphocytes associated neutropenia have been described, including a familial form [151].

### Ethnic neutropenia

Ethnic neutropenia is the most frequent form of chronic neutropenia. It is generally isolated and moderate, and has no direct health repercussions. The mode of genetic transmission is not yet known and may be multifactorial. First described in 1941 [152], it appears to be particularly frequent in black-skinned individuals. Epidemiological studies show that the prevalence of neutropenia (<1.5 G/l) is about 4.5% in black people and 0.8% in Causasians [153]. Few data are available on other populations, but a high frequency has been noted in the Arabian peninsula [154], and the frequent mild neutropenia reported in Crete likely corresponds to the same entity [155].

Ethnic neutropenia is not associated with increased susceptibility to infections, and no symptoms have ever been reported.

Four simple but poorly specific features are classically present: moderate neutropenia (0.5 to 1.5 G/l), no infections attributable to neutropenia, no identifiable cause, and African ethnicity.

The few available studies of ethnic neutropenia have yielded strictly normal findings. In particular, the bone marrow is qualitatively and quantitatively normal. No difference in neutrophil mobilization after brief or more strenuous exercise (such as marathon running) has been observed, and this form of neutropenia does not appear to be due to excessive neutrophil margination [156]. A particular polymorphism of the Duffy antigen receptor for cytokines (DARC) [157] is associated with ethnic neutropenia in black people.

## Antenatal diagnosis

Monogenic congenital neutropenias are severe diseases with a sometimes major impact on the patient's daily life; antenatal diagnosis is therefore warranted, taking into account the mode of transmission [158].

Antenatal diagnosis has already been performed for the following disorders: severe congenital neutropenia with pathogenic *ELANE *mutations, Shwachman-Diamond syndrome, WHIM syndrome and glycogen storage disease type Ib and may be offered for many other entities, such as *HAX1 *and *G6PC3*.

Antenatal diagnosis must be preceded by a familial survey. Neutropenia due to mutation of the *ELANE *gene, which shows autosomal dominant transmission, can exist in the mosaic state in one of the parents, with a unusually high frequency compared with other genetic abnormalities [159].

The decision to implement antenatal diagnosis and, possibly, pregnancy termination, must be taken within a precise ethical framework, after providing the family with thorough information and obtaining medical expert opinion in each individual case.

## Treatment and Patient Management

### Historical perspective

Until the 1960s, children with severe congenital neutropenia had a grim prognosis [2,30]. Survival started to improve markedly in the 1970s, thanks to progress in curative parenteral antimicrobial chemotherapy and to more general use of antibiotic prophylaxis. However, quality of life remained mediocre because of recurrent infections and permanent stomatitis.

With the exception of bone marrow transplantation[160], no other treatment (steroids, levamisole, lithium, etc.) was able to correct the neutropenia. Hematopoietic growth factors (G-CSF and GM-CSF) started to be used in 1988 [161], and immediately proved capable of correcting both the neutropenia and susceptibility to infections. The arrival of these drugs also kindled interest in these disorders, leading to the creation of registries in several western countries.

### Management of acute infectious episodes

It is important to assess the potential gravity of each infectious episode by means of a thorough physical examination. Precious information on the risk associated with bacterial infections can be gleaned from experience in cancer chemotherapy, which shows the importance of body temperature (>39°C) and the decline in the monocyte count (<0.1 G/l) as gravity factors [162,163].

In moderate neutropenia complicated by superficial or ENT infections, oral antibiotic therapy is adequate, with close ambulatory monitoring, if inflammatory markers are absent (e.g. CRP < 15 mg/L).

In contrast, patients with severe neutropenia and sepsis require immediate hospitalization [164]. After bacteriological examinations (blood culture, urinalysis, local samples, etc.) and chest radiography, parenteral antibiotic therapy should be started rapidly, with a combination of a third-generation cephalosporin and an aminoside by IV route. The place of glycopeptides (vancomycin and teicoplanin) in first-line therapy is controversial. In fever persists beyond 48 hours, antifungal treatment should be added.

If the child's condition is worrisome from the outset, G-CSF administration should be started, either at a dose to which the patient is known to respond, or at the standard dose of 5 μg/kg/d. The dose should be increased if no improvement is observed. This is justified even when the precise etiology of the neutropenia is not known. There is no reason to suspect that temporary G-CSF administration would hinder subsequent investigations.

The utility of leukocyte concentrates should be underlined, even if their use is now generally limited to cellulitis and documented bacterial or fungal infections showing no clinical response to appropriate antibiotic therapy.

### Prevention of infections

It is crucial to prevent recurrent infections. The use of antimicrobial prophylaxis depends on the individual risk, history, and severity of neutropenia.

#### Antimicrobial prophylaxis

The principal measure is antimicrobial chemoprophylaxis. The ideal antimicrobial chemotherapy regimen will be effective against the pathogens most frequently encountered in this setting, well tolerated, and not select resistant strains. On this basis the best antibiotic is the oral sulfamethoxazole/trimethoprim combination (Bactrim^®^), at a daily dose of 50 mg/kg per day. Its value in constitutional neutropenia has not been studied, but it is reasonable to extrapolate data from patients with leukemia [165] and chronic granulomatous disease (CGD) [166]. The use of this drug in chronic neutropenia sometimes appears paradoxical, because it can occasionally cause neutropenia [167]. However, the risk-benefit ratio remains favorable. Sulfamethoxazole/trimethoprim only partially prevents these patients' gingivostomatitis, and concurrent therapy with an agent active on the oral saprophytic flora, and especially anaerobes (metronidazole), is therefore warranted.

#### Use of cytokines in constitutional neutropenia

The second possibility is to correct the neutropenia by using hematopoietic growth factors (G-CSF and GM-CSF) produced by genetic engineering.

In practice, only G-CSF is used in this indication [42]. Indeed, GM-CSF has several disadvantages, with lesser efficacy in these indications and poorer immediate tolerability ('flu-like syndrome, marked eosinophilia) [168-170]. G-CSF is currently available in two forms: filgrastim (Neupogen, in vials containing 480 μg or 330 μg) and lenograstim (Granocyte, in vials containing 340 μg or 130 μg). These two molecules are nearly identical, lenograstim being the glycosylated form of G-CSF. Their biological effects are also practically the same, but one study suggests that filgrastim yields a slightly larger increase in the neutrophil count compared with the same dose of lenograstim [171]. It is important to underline that the pegylated form of G-CSF (PegFilgrastim Neulasta^®^) is not registered for patients with congenital neutropenia. Pegfilgrastim -- a combination of filgrastim and polyethylene glycol -- has a half-life of 15 to 80 hours, reducing the required number of injections [172]. However, pending specific pharmacokinetic data in congenital neutropenia, its use carries a risk of overdose and potentially severe adverse effects [173], or, on the contrary, a lack of efficacy [174].

##### Treatment schedule

In severe congenital neutropenia, the time and the dose required to increase the neutrophil count cannot be predicted. In other indications the schedule is generally simpler. Long-term treatment takes place in two phases [175]: an induction phase and a maintenance phase. The aim of the induction phase is to characterize the individual response to G-CSF. The response is evaluated in terms of the increase in the neutrophil count (> 1.5 G/l) and the clinical improvement, after 10 to 15 days. Serial blood cell counts are useful for following changes in the neutrophil count during this period.

The recommended initial daily dose is 5 μg/kg subcutaneously, with no particular constraints regarding the timing of injections during the day. If no response is seen after 15 days, the daily dose is increased in steps of 5 μg/kg. On the contrary, if the response is rapid or even excessive (PN > 5 G/l), the dose should be halved. Short-term tolerability is also assessed during the induction phase, including dose-dependent adverse effects that will have to be taken into account during long-term treatment. Once the minimal daily dose has been determined, the maintenance phase can begin, which aims to determine the minimal dose and rhythm of injection to sustain a clinical response. In the maintenance phase it is of course possible to modulate the dose and sometimes to attempt a dose reduction or a longer dosing interval. But it may be necessary to increase the daily dose, especially for a growing child. Unnecessary blood counts should be avoided during this period: unless clinical problems arise, a monitoring interval of 4 to 6 months is acceptable.

##### Efficacy

##### Severe congenital neutropenia

Between 1988 and 2010, the international and French registries collated data on G-CSF therapy in no more than 500 patients with severe congenital neutropenia [6,21]. Long-term follow-up confirms the results of short phase I/II trials in small groups of patients [175,176]. The efficacy of G-CSF is based first on the neutrophil count. It is now clear that the response does not wane with time. However, the most important criterion for efficacy is the reduction in infectious complications, including stomatologic status, although there are no well-established criteria for this endpoint. During G-CSF development only one randomized study, involving 36 patients, focused on infections [176]. In this study, some patients received G-CSF routinely and others only after a 4-month observation period. This study demonstrated a benefit in terms of both infections and quality of life. No long-term randomized trials have been published. The dose required to obtain a response varies widely from one patient to another. Almost two-thirds of patients respond to a daily dose of between 2 and 10 μg/kg, while nearly 20% respond to 10-20 μg/kg. A small number of patients require even higher doses of up to 100 μg/kg. Only 13 cases of complete G-CSF treatment failure have been reported [6,21]

The neutrophil count increment is dose-dependent beyond a minimum threshold, but it fluctuates over time on a stable dose, with no identifiable pattern.

There are no clinical or biological features predictive of the dose to which a given patient will respond.

##### Cyclic neutropenia

G-CSF is always effective in this setting, avoiding the neutrophil nadir. In contrast, it does not abolish the cyclic nature of granulopoeisis, and the oscillations tend to be more unstable, with peak counts sometimes exceed 30 G/l. Despite several attempts, no cyclic schedule of G-CSF administration, such as once every third week, has proven effective. In contrast, the dose required to correct the nadir is generally below 5 μg/kg/d and the injections can be given intermittently, for example once every three days.

##### Tolerability of G-CSF

##### Short-term adverse effects

G-CSF has been used briefly (<15 days), at a dose of 1 to 5 μg/kg/d, in more than a million cancer patients (children and adults) receiving chemotherapy. Tolerability during such short-term use is good or excellent. Both intravenous and subcutaneous injections provoke only occasional immediate (<1 per 100) and local reactions. 'Flu-like reactions, as observed with other cytokines, are also infrequent.

Bone pain is more frequent, affecting 2 to 5% of subjects. It rapidly subsides on treatment cessation (within less than 24 hours) and generally does not recur when a lower dose is adopted.

##### Long-term tolerability

Few situations necessitate long-term G-CSF therapy. In addition to chronic neutropenia, G-CSF is sometimes administered in the long term for aplastic anemia. Published reports of long-term G-CSF safety concern fewer than 1500 patients, with variable levels of drug exposure [6,21,177]. Although the action of G-CSF is, in principle, limited to the granulocyte lineage, various hematologic abnormalities can be present or occur transiently during treatment. Monocytosis beyond 1.5 G/l is frequent. Eosinophilia, frequent at diagnosis, can be amplified by G-CSF. Lymphocytosis is unaffected, as is the hemoglobin level in most patients. However, reticulocytosis occasionally increases, along with the hemoglobin level, especially if inflammatory anemia is present at the outset of treatment. Thrombocytopenia seems to be the most common hematologic adverse effect. However, it is generally moderate and regresses when the G-CSF dose is reduced. Thrombocytopenia can also be due to hypersplenism [177]. The spleen almost always enlarges (on imaging studies) at the outset of treatment. Clinical confirmation of this splenomegaly is rarely obtained, except in glycogen storage disease Ib, in which this complication is very frequent. Spleen rupture necessitating splenectomy can occasionally occur [177]. The uricemia rises during long-term treatment but this has no clinical consequences. Exacerbation of long-standing gout has been observed during short-course therapy [178]. The first cases of leukocytoclasic vascularitis, corresponding to Sweet's syndrome, were observed after short-term treatment (<1 month)[179]. The G-CSF-induced increase in neutrophil adhesion molecule expression seems to be responsible. These cutaneous manifestations always regress after a dose reduction or treatment cessation. Two cases of mesangioproliferative glomerulonephritis have been reported during long-term treatment, both resolving after a dose reduction or treatment cessation. Osteoporosis occurs in nearly one-quarter of patients with severe congenital neutropenia who receive chronic G-CSF therapy [180]. Two cases of pathological fracture have been reported. But, severe congenital neutropenia itself seems to be associated with osteopenia, which is often present before treatment begins. Somatic development is unaffected by G-CSF and so is puberty.

##### Multidisciplinary management of multisystem disorders

##### Glycogen storage disease Ib

Glycogen storage disease Ib patients must be managed by a multidisciplinary team including experienced specialists in pediatric nutrition and hematology. The carbohydrate balance is very fragile in these patients, who must be fed day and night. Severe carbohydrate instability may necessitate liver transplantation [68]. Low doses of G-CSF (< 5 μg/kg/d) are usually necessary both to correct the neutropenia and to obtain a clinical improvement [21,181]. The response is obtained within 48 hours, which is compatible with neutrophil release from the bone marrow compartment and with the absence of maturation arrest in these patients. Tolerability is generally good, although G-CSF-induced thrombocytopenia and splenomegalia are relatively frequent.

##### Shwachman-Diamond syndrome

These patients too must be managed by a multidisciplinary team. Their external pancreatic insufficiency leads to nutritional deficiency, which may be severe or simply consist of liposoluble vitamin deficiencies, requiring supplementation. Attention must be paid to the accompanying bone disorders, and counseling is needed because of these patients' frequently abnormal mental development. The use of G-CSF is far less frequent in patients with permanent *ELANE *neutropenia.

#### Hematopoietic stem cell transplantation (HSCT)

HSCT can permanently correct the neutropenia in these patients, and is the only option for patients who continue to experience severe infections despite G-CSF therapy.

Validated indications of bone marrow grafting include G-CSF resistance (> 50 μg/kg/day) and myelodysplasia/leukemic transformation, in which case it is the only therapeutic option [182-184].

Patients with malignant transformation (with the exception of frank leukemia) should not receive chemotherapy before the bone marrow graft.

In patients with neutropenia dependent on chronically high doses of G-CSF (at least 20 μg/kg per injection at least three months a year), given the high risk of leukemic transformation, bone marrow grafting should be considered on a case by case basis, taking into account the possibility of finding a related donor.

Standard HSCT procedures can be used, with myeloablative conditioning. Even in patients with malignant transformation survival now exceeds 70%, with the exception of patients with Schwachman's syndrome.

The second disease in which HSCT may be indicated is Shwachman-Diamond syndrome. Schematically, there are two distinct indications for HSCT in this setting: pancytopenia with no detectable malignant clone, and myelodysplasia/leukemic transformation [185]. The results are very different in the two indications but tend to be good (favorable outcome in >80% of cases) in the absence of a malignant clone, while they are very mediocre in case of leukemic transformation (<35% survival). In the absence of clonal progression, the so-called 'reduced intensity' conditioning regimen (especially fludarabine and Campath) appears promising [186,187].

### Daily life

It should be remembered that intramuscular injections and rectal temperature measurement may be harmful. Most vaccines can be administered, including live viral vaccines. However, BCG vaccine should be avoided. Pneumococcal and influenza vaccination is recommended. No dietary restrictions are necessary in neutropenic children. They are not unusually susceptible to viral epidemics and there is therefore no reason to deprive them of opportunities for social interaction.

## Prognosis and Outcome

Several complications can occur in patients with congenital neutropenia, including infectious complications, complications related to extra-hematological involvement, and the risk of leukemia related both to the disease and its treatment.

### Outcome - the infectious risk

Bacterial infections represent the main risk. Infections can be life-threatening or otherwise impair quality of life. This is particularly the case of chronic oral infections, leading to recurrent aphthosis, paradontopathy and tooth loss.

The natural risk of life-threatening invasive infections is very high. In the 1950s, almost all patients with the most severe form of the disease, with permanent and profound neutropenia, died in the first 2 years of life from sepsis, cellulitis or pneumonia; this was the case of 11 of the 14 patients in Kostmann's pedigree [2]. Two deaths from pneumonia were reported among 16 patients with cyclic neutropenia [188], while no deaths were reported among patients with chronic benign neutropenia [34,142].

In the sixties and seventies, with more extensive used of antibiotic therapy, lethal sepsis became less frequent even in the most severe forms of congenital neutropenia. The report of Kostmann's pedigree in 1975 showed long-term survival [33] and prior the G-CSF period (since the ninety's) death from infections is already exceptional in such category of patients but occasionally is described even cyclic neutropenia [5].

Chronic infections remain very frequent, and especially stomatologic infections with painful gingivitis associated with papules (aphthae-like oral furuncles) of the tongue, and parodontopathies [35]. Diffuse gastrointestinal lesions are sometimes present, leading to abdominal pain and diarrhea, and sometimes mimicking Crohn's disease on radiological studies [36]. This complication is frequent in glycogen storage disease type Ib.

The availability of G-CSF since 1988 dramatically changed these patients' medical management, but lethal bacterial infections are still reported [21,189], especially in patients with a poor response to G-CSF, or with poor compliance. However, chronic stomatologic infection remains very difficult to manage, even with G-CSF and neutrophil recovery, leading to tooth loss [190]. Finally, the infections risk may not be related only to the neutropenia: the best example is the WHIM syndrome, which combines lymphopenia, hypogammaglobulinemia and very high susceptibility to human papillovirus infections [191].

### Outcome: morbidity related to extra-hematopoietic involvement

Extra-hematopoetic involvement may have a very strong impact on these patients' lives, such as the neurodevelopmental disorders observed in Kostmann's disease, Shwachman Diamond syndrome, and Cohen's disease. Cardiac dysfunction may be very severe in Shwachman Diamond syndrome and is almost always observed in Barth's syndrome.

### Malignant transformation: Risk factors and possible role of G-CSF

First introduced in the late 1980s [42], growth factors have vastly improved the management of chronic neutropenia. Once their efficacy on the neutropenia associated with cancer chemotherapy had been demonstrated [161] and the need for long-term administration in some cases had emerged [176], questions of safety were raised, especially regarding the risk of malignant transformation.

Although congenital neutropenias are preleukemic states, the risk of malignant transformation is difficult to evaluate in isolation, as the spontaneous risk and the potential role of G-CSF must both be taken into account.

The main question is the risk-benefit ratio, as mentioned in the very first article reporting the effect of G-CSF in this setting [161], particularly as leukemias had been observed in the rare patients with congenital neutropenia who survived beyond their first decade of life [192-194].

In 1993 international teams opted to create a patient registry to examine this issue. The data confirmed the marked increase in the risk of leukemia in these patients. The cumulative incidence of leukemia among patients with severe congenital neutropenia is about 15% at age 20 years [21].

Leukemic transformation has been observed in patients with mutations in *ELANE *[21,195], *HAX1 *[196], *WASP *[91], *SBDS *[21,197] and *G6PC3 *or *SLC37A4 *[198,199].

These leukemias have a number of particularities. They usually involve proliferation of poorly differentiated cells, and the most consistent cytogenetic anomaly is monosomy 7. They are often preceded by the emergence of an acquired somatic anomaly of the intracytoplasmic part of the G-CSF receptor CSF3R [200]. These mutations are not found in de novo acute myeloblastic or lymphoblastic leukemia [201] and have never been found in Shwachman Diamond disease, even though this entity is associated with a high risk of leukemic transformation.

Studies of patients with *ELANE *mutations show that the main risk factor for leukemic onset is the severity of neutropenia and not the nature of the *ELANE *mutation. Thus, the leukemic risk is very low or inexistent in cyclic neutropenia, while it is maximal in patients with permanent neutropenia below 0.1 G/l. However, several other factors are associated with severe neutropenia, such as infections and the use of G-CSF, especially at high doses (> 15 μg/kg/day) and for long periods[21].

The link between intracytoplasmic G-CSF receptor defects, often observed in these patients before and during malignant transformation[202,203], and monosomy 7 is not known. In contrast, blast cell lineages harboring monosomy 7 are particularly sensitive to G-CSF, which selects cells carrying this anomaly [204]. The absence of any direct oncogenic effect of *ELANE *mutations and the impact of both the severity of neutropenia (that strongly modifies bone marrow homeostasis and leads to compensatory hyperstimulation of the monocyte lineage) and high doses of G-CSF suggests that long-term bone marrow stimulation can be responsible for leukemic transformation. The leukemic risk in patients receiving the highest doses of G-CSF may warrant HSCT [21,182,189].

#### Evaluation of the risk factors of secondary leukemia with patient registries

The number of reported cases of leukemia and myelodysplasia in patients with severe congenital neutropenia has increased markedly in recent years, since the advent of G-CSF [6,7,205-210] relative to the pre-G-CSF era [192-194]. Numerous cases have been described in patients with Shwachman-Diamond syndrome, both before the advent of G-CSF and also in patients not receiving this drug [60,211-213], whereas few have been reported in patients receiving G-CSF. However, this syndrome is rarely treated with G-CSF.

Reviews of the literature provide less reliable information on this adverse effect than patient registries, which can be used to calculate and to compare the risk.

The risk of leukemia and myelodysplasia has been studied in the French registry [21]. Factors favoring malignant transformation included disease-related factors and G-CSF exposure. Disease-related factors comprise the type of neutropenia as myelodysplasia and leukemia are observed only in patients with severe congenital neutropenia or Shwachman Diamond syndrome, the severity of neutropenia (i.e. the number and severity of infections), the degree of neutropenia, and the level of bone marrow myeloid arrest. Two characteristics of G-CSF exposure are significantly linked to the risk of leukemic transformation: the cumulative dose and the mean dose per injection. The cumulative duration of G-CSF exposure and the length of post-treatment follow-up are not associated with an increased risk. No threshold of exposure below which G-CSF does not increase the leukemic risk has been identified. In addition, the small size of this sample rules out firm conclusions, but patients requiring more than 10 μg/kg per injection and who receive a cumulative dose of more than 10 000 μg/kg, clearly have an increased risk of malignant transformation.

Initial publications from the international registry did not examine the link between the intensity of G-CSF exposure and the leukemic risk [6,7], but more recent analyses have shown an increase in the risk of leukemia among patients receiving the highest doses [189,214].

A relation between G-CSF exposure and secondary myelodysplasia and leukemia have been obtained in cohort studies of patients with bone marrow aplasia [215,216] and breast cancer [217-219], showing that G-CSF has a leukemogenic effect in situations clearly distinct from congenital neutropenia.

#### Monitoring of the leukemic risk

This leukemic risk warrants close patient monitoring, especially when high doses of G-CSF are used. Repeated blood cell counts are required to detect anemia or thrombocytopenia, which may necessitate bone marrow sampling.

The place of routine bone marrow sampling is more controversial. Intracytoplasmic G-CSF- receptor mutations correlate with the appearance of leukemic clones, which can also be detected by cytogenetic examination with the FISH technique.

#### Conclusion on the leukemic risk of G-CSF therapy

Although the data are somewhat fragmentary and heterogeneous, several clinical studies suggest that G-CSF exposure beyond a certain threshold can be leukemogenic in patents with disorders known to favor leukemic transformation. The precise threshold dose at which this effect emerges cannot be determined, as it is not mentioned in most studies.

## Lessons from congenital neutropenia and perspectives

Knowledge of the molecular bases of congenital neutropenia provides important information on two aspects of myeloid differentiation.

### How congenital neutropenia contribute to understand dynamics of granulopoeisis?

The link between permanent neutropenia and defective neutrophil production or excessive apoptosis of neutrophil precursors is clear: a decrease in the production of cells or shorter half-life results in a lower number in the periphery. The cyclic aspect of the peripheral neutrophil count is more difficult to analyze and suggests the existence of a cryptic biological clock that regulates granulopoiesis. This putative clock might be revealed by particular mutations. To unify cyclic and permanent neutropenia, older notions on the dynamics of granulopoiesis can be helpful. First, the neutrophil count in healthy subjects varies markedly (between 1.8 and 4.5 G/l), in an unpredictable and chaotic manner [220]. In addition, 'extrinsic' factors that affect the neutrophil count also modify the cyclic variations in circulating neutrophil numbers relative to healthy subjects. Thus, G-CSF not only increases the number of circulating neutrophils but also leads to pseudo-cyclic irregularities [161]. In contrast, a cytostatic drug such as cyclophosphamide, administered at low doses, transforms chaotic variations in the neutrophil count into pseudo-cyclic changes[221], while high doses cause profound permanent neutropenia. These phenomena correspond to a non linear mathematical model [222]. Such models can describe temporal variations in the size of a population as a chaotic variation, with no precise cycle but between permanent extremes, up to total abolition of the population, via quasi-sinusoidal variations, from variations in a single coefficient corresponding to the reproductive rate of the population, i.e. the relationship between the production and death of individuals composing the population. In the case of the neutrophil population, excessive apoptosis, that cannot be precisely quantified and that can be influenced by precise mutations and the epigenetic context [223], contributes to excessive cell death [224]. This model remains theoretical but can be used to integrate physiological and pathological situations affecting granulopoiesis and is the only way to unify the different situations observed in congenital neutropenia[19].

### The fate of stem cells from immature myeloid cells to mature polymorphonuclear neutrophils. lessons from congenital neutropenia

Congenital neutropenia represents a physiological model for studying granulopoeisis. In the past 10 years, 12 genes responsible for congenital neutropenia have been identified. Each mutation is responsible for a very peculiar molecular defect. Surprisingly, most known molecular abnormalities responsible for neutropenia do not involve genes with a transcriptional role in granulopoeisis, but rather genes involved in endoplasmic reticulum functions, like granule stability or intracytoplasmic granule trafficking or protein packaging.

Defective packaging of cellular enzymes in granules (due to *ELANE *mutations) or cytoskeleton changes (*WASP *and *dynamin 2 *mutations) modify intracytoplasmic trafficking and result in neutropenia, possibly through an excess of apoptosis or defective maturation. This is similar to the situation in several clinical disorders comprising albinism and neutropenia and characteristic of the Hermansky-Pudlak syndrome type 2 (*AP3 *defect), *AP14 *deficiency [78] (AP14 is a protein with similar functions to *AP3*), Chediak-Higashi syndrome and Griscelli's syndrome (the latter two entities also involve a macrophage activation syndrome in addition to neutropenia) [114]. A transmembrane protein of the Golgi apparatus is also involved in other disorders comprising neutropenia, such as glycogen storage disease Ib (*SLC37A4*), G6PC3 and Cohen's disease, but whose phenotypic expression also involves other systems.

The involvement of endoplasmic reticulum (ER) proteins or ER packaging processes in these forms of neutropenia shows the importance of ER stress. Increased ER stress elicits a cellular response known as the unfolded protein response (UPR). The UPR is activated in response to an accumulation of unfolded or misfolded proteins in the lumen of the ER. In this scenario, the UPR has two primary aims: to restore normal cell function by halting protein translation and to activate the signaling pathways that lead to increased production of molecular chaperones involved in protein folding. If these objectives are not achieved within a certain time lapse or if the disruption is prolonged, the UPR initiates programmed cell death (apoptosis). Three ER-localized protein sensors are known: IRE1alpha (inositol-requiring 1alpha), PERK (Protein kinase (PKR)-like ER kinase), and activating transcription factor 6 (ATF6). In cases of ER stress, these sensors are activated and trigger a complex series of events designed to maintain ER homeostasis and to promote protein folding, maturation, secretion, and ER-associated protein degradation. Changes in the shape of the protein, or a change in its function - such as a gain of function - may be one trigger of ER stress as shown for *ELANE *mutations [48,51]. No comprehensive mechanism has been proposed for the impact of several different mutations, such as *SBDS *[225]. Indeed, mutation of the only transcription factor (GFI1) known to be involved in congenital neutropenia may not directly affect the transcription process but interfere with interaction with *ELANE *protein [88,226].

Lymphoid enhancer factor 1 (LEF1) is a 48-kD nuclear protein expressed in pre-B and T cells and in myeloid cells. A low level of this factor has been observed in the myeloid cells of patients with congenital neutropenia with maturation arrest at the promyelocyte level [227]. The decrease in transcription factor expression is difficult to interpret both because *LEF1 *gene is normal and because LEF1 expression is depending of type of congenital neutropenia. This suggests that the decreased expression of LEF1 is more a proteic consequence to some mutations causing congenital neutropenia. Indeed Horwicz's team, who initially showed the involvement of LEF1 in this pathway, have shown that LEF-1 cooperates with Core-binding Factor α to activate *ELANE *in vivo [228]. They also raised the possibility that up regulating promoter mutations may contribute to SCN.

Pro LL37 is a antibacterial peptide usually packaged in neutrophil granules and its level is low, whatever the genetic backgrounds of the congenital neutropenia [229]. Low pro LL37 level may be responsible for the persistence of parondothopathy in patients with CN treated with G-CSF. Interestingly, vitamin D is able to correct the pro LL37 level [230].

Vitamin B3 (nicotamide) participates in a regulatory loop controlling the transcriptional expression of G-CSF. Vitamin B3 induces a peripheral increase in neutrophils [231].

## Competing interests

The authors declare that they have no competing interests.

## Authors' contributions

JD conceived the design and the first draft of the manuscript. OF collect the cytological iconography, all authors critically review the initial draft and participate to the final version of the manuscript.

## Supplementary Material

Additional file 1 **Plate #1: Large aphthae on inner lip of a patient with severe congenital neutropenia**. Plate #2: Inflammatory gum lesion in a 12-y-old body with severe congenital neutropenia. Note the enamel damage and loss of parodontal tissue. Plate #3: Aspects of maturation arrest at the promyelocytic stage associated with hypereosinophilia and monocytosis in a patient with *ELANE *severe congenital neutropenia. Plate #4: Marrow smear in a patients with Shwachman-Diamond syndrome complicated by bone marrow aplasia: Poor cellularity, fat cells and mast cells. Plate #5: Marrow smear in a patient with Shwachman-Diamond syndrome complicated by acute erytrhoid leukemia. Plate #6: Marrow smear in a patient with Shwachman-Diamond syndrome complicated by cytopenia and monosomy 7. Left: monolobated micromegacaryocyte (arrow) Right: double nucleus of the granulocyte lineage (arrow). Plate #7: Marrow smear in a patient with glycogen storage disease 1b: Hyperplasia of the granulocyte lineage with no maturation arrest. Plate #8: Marrow smear of a patient with WHIM syndrome: The PN nuclear lobes are separated by long, thin filaments; the cytoplasm is occasionally vacuolated. Plate #9: Blood smear of a patient with Chediak-Higashi Syndrome: Left: lymphocyte with a voluminous bright red inclusion (MGG staining) Middle: PN with large sparse granulation, Right Marrow smear of a patient with Chediak Higashi Syndrome: Voluminous inclusions in the cytoplasm of myeloid precursors. Plate #10: Marrow smear of a patient with Griscelli Syndrome: Numerous histiocytes reflecting a histiocyte activation syndrome. Plate #11: Marrow smear of a patient with dibasic protein intolerance: Left: PN with picnotic nuclei phagocyted, by immatures myeloid cells (centre) and by histiocytes (right). Plate #12: Marrow smear of a patient with Pearson's syndrome: Vacuolization of precursors (left) associated with dyserythropoiesis with acidophilic cells with laminated cytoplasm (centre) and ring sideroblasts (Perls stain) (right). Plate #13: Marrow smear in a young patient with autoimmune neutropenia Left: Phagocytosis of a neutrophil by a histiocyte. Right: Two histiocytes having engulfed several neutrophils that are at various stages of breakdown Click here for file
