# Supplementary material for: Congenital neutropenia: diagnosis, molecular bases and patient management
Source: Orphanet J Rare Dis. 2011 May 19;6:26. doi: 10.1186/1750-1172-6-26 (PMC3127744; doi:10.1186/1750-1172-6-26)
Supplement: Additional file 1 — Plate #1: Large aphthae on inner lip of a patient with severe congenital neutropenia. Plate #2: Inflammatory gum lesion in a 12-y-old body with severe congenital neutropenia. Note the enamel damage and loss of parodontal tissue. Plate #3: Aspects of maturation arrest at the promyelocytic stage associated with hypereosinophilia and monocytosis in a patient with ELANE severe congenital neutropenia. Plate #4: Marrow smear in a patients with Shwachman-Diamond syndrome complicated by bone marrow aplasia: Poor cellularity, fat cells and mast cells. Plate #5: Marrow smear in a patient with Shwachman-Diamond syndrome complicated by acute erytrhoid leukemia. Plate #6: Marrow smear in a patient with Shwachman-Diamond syndrome complicated by cytopenia and monosomy 7. Left: monolobated micromegacaryocyte (arrow) Right: double nucleus of the granulocyte lineage (arrow). Plate #7: Marrow smear in a patient with glycogen storage disease 1b: Hyperplasia of the granulocyte lineage with no maturation arrest. Plate #8: Marrow smear of a patient with WHIM syndrome: The PN nuclear lobes are separated by long, thin filaments; the cytoplasm is occasionally vacuolated. Plate #9: Blood smear of a patient with Chediak-Higashi Syndrome: Left: lymphocyte with a voluminous bright red inclusion (MGG staining) Middle: PN with large sparse granulation, Right Marrow smear of a patient with Chediak Higashi Syndrome: Voluminous inclusions in the cytoplasm of myeloid precursors. Plate #10: Marrow smear of a patient with Griscelli Syndrome: Numerous histiocytes reflecting a histiocyte activation syndrome. Plate #11: Marrow smear of a patient with dibasic protein intolerance: Left: PN with picnotic nuclei phagocyted, by immatures myeloid cells (centre) and by histiocytes (right). Plate #12: Marrow smear of a patient with Pearson's syndrome: Vacuolization of precursors (left) associated with dyserythropoiesis with acidophilic cells with laminated cytoplasm (centre) and ring sideroblasts (Perls stain) (ri [file 1750-1172-6-26-S1.PPT]

## Slide 1
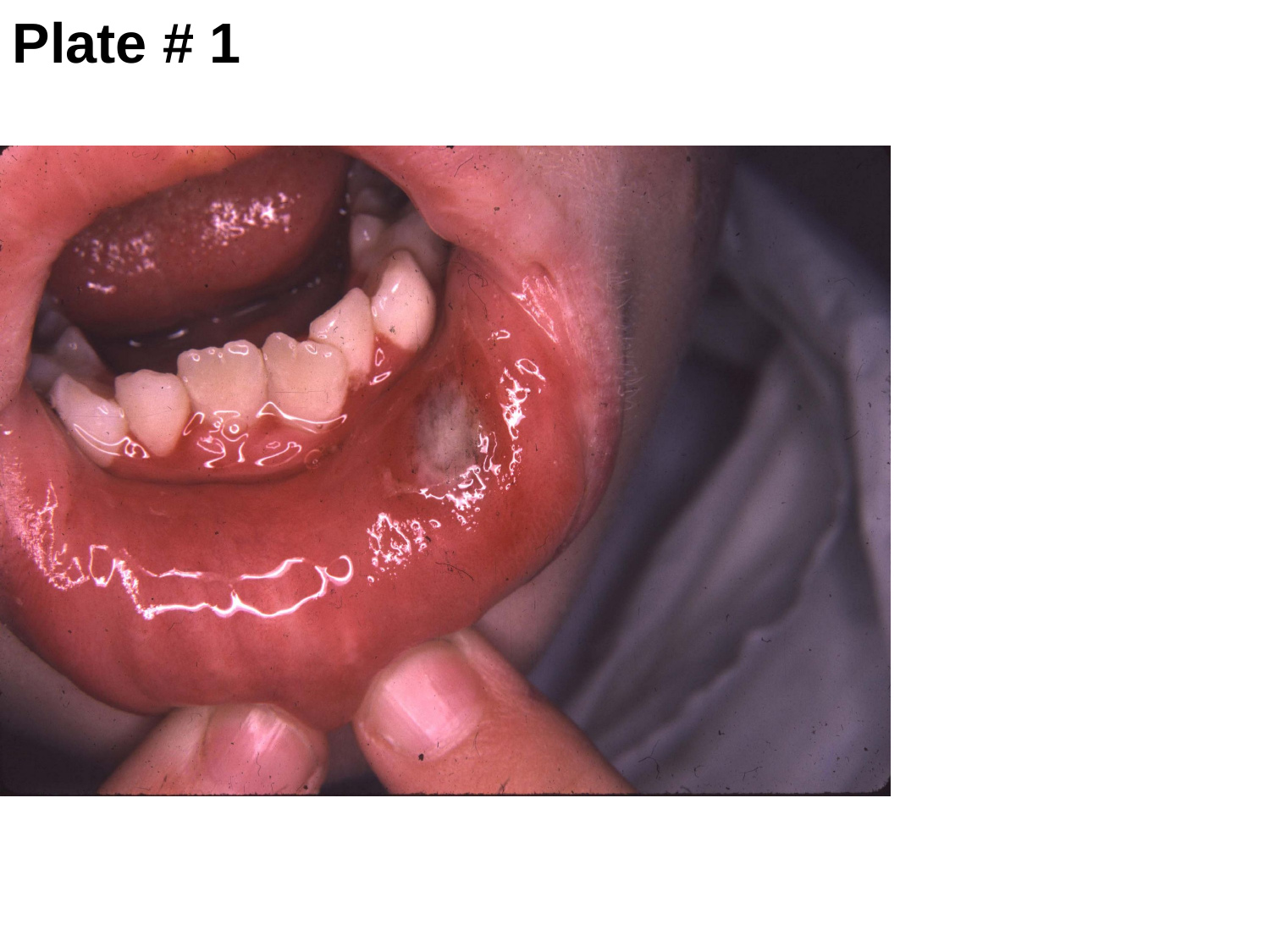

Plate # 1

## Slide 2
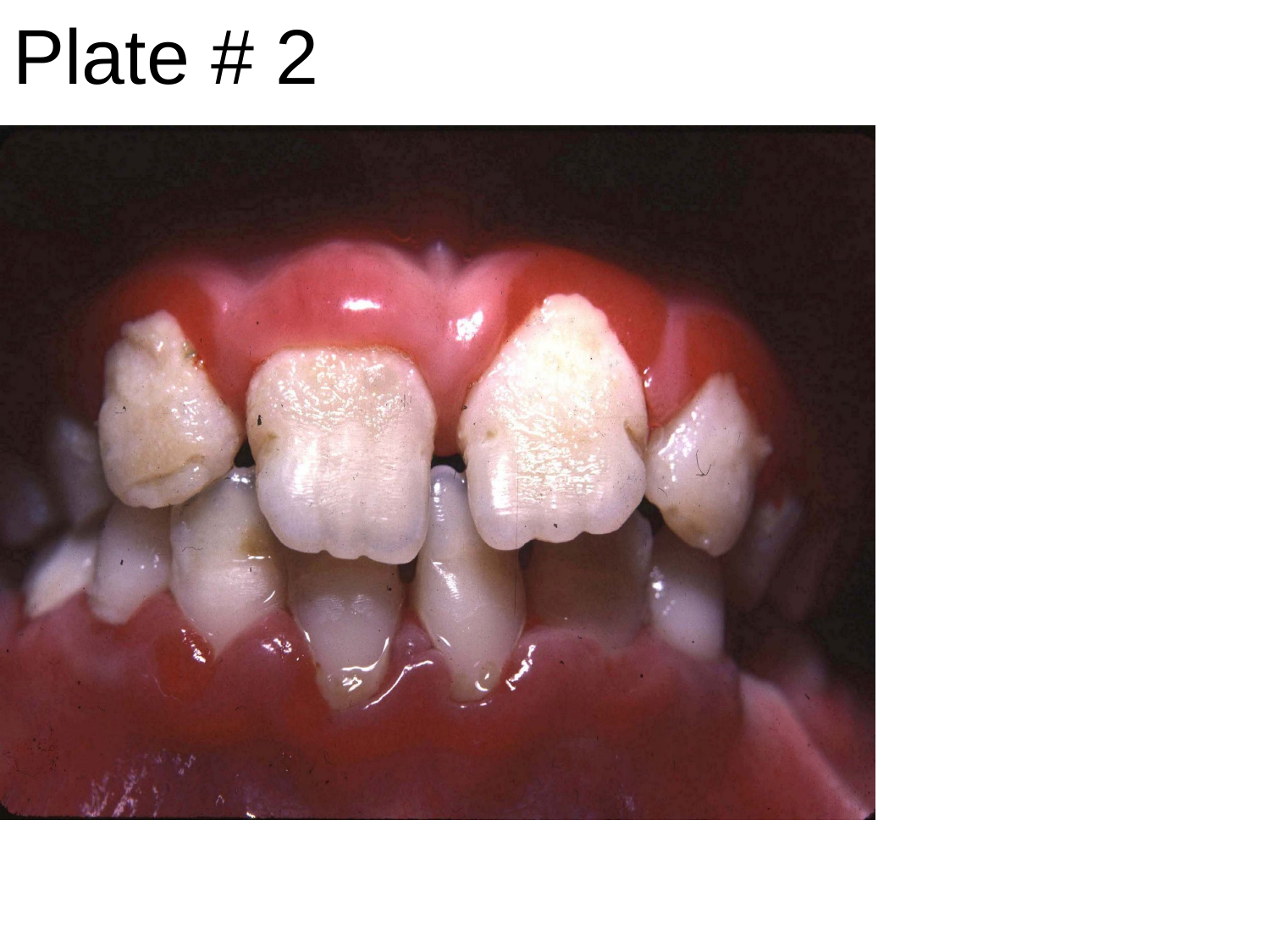

Plate # 2

## Slide 3
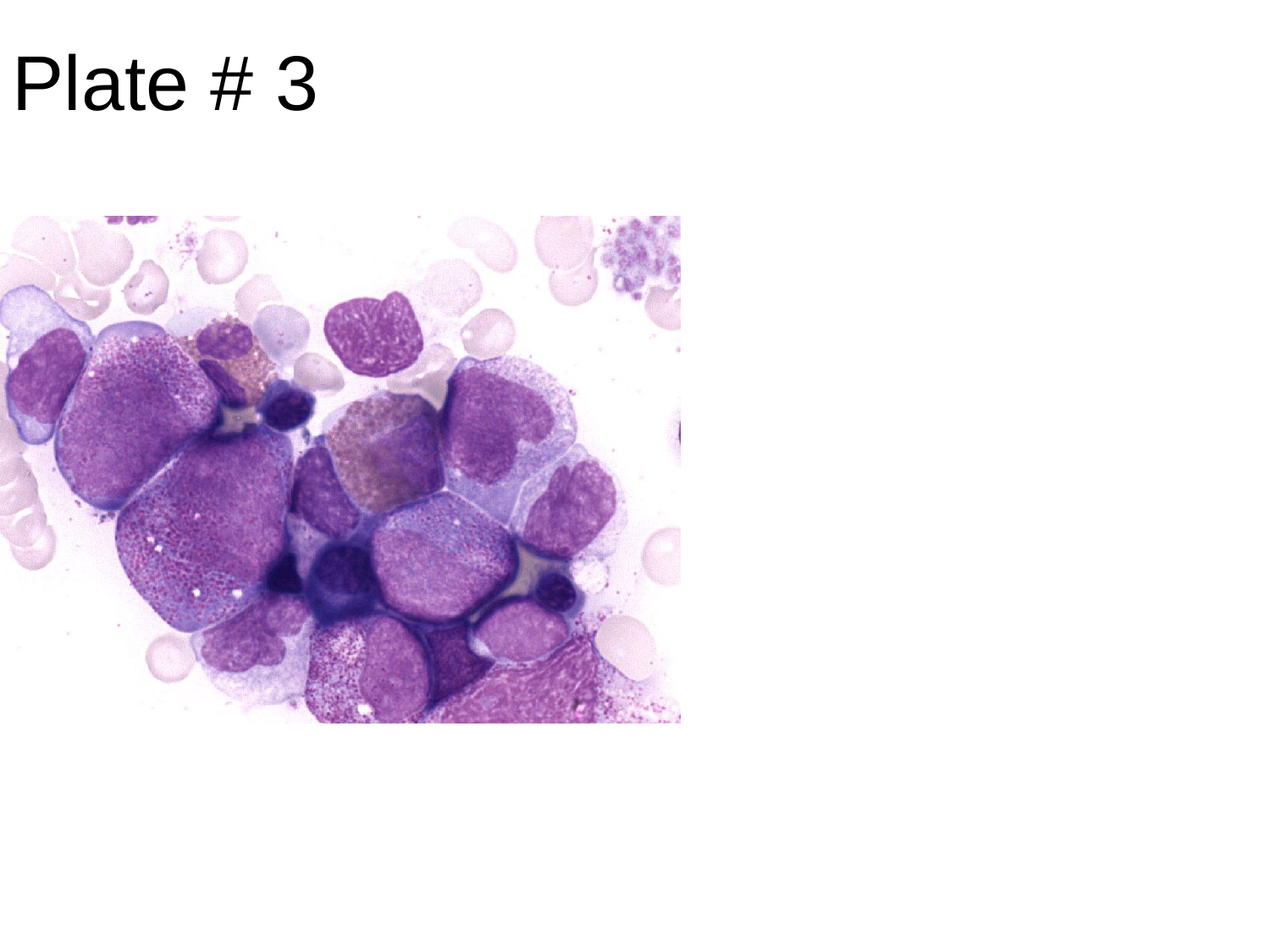

# Plate # 3

## Slide 4
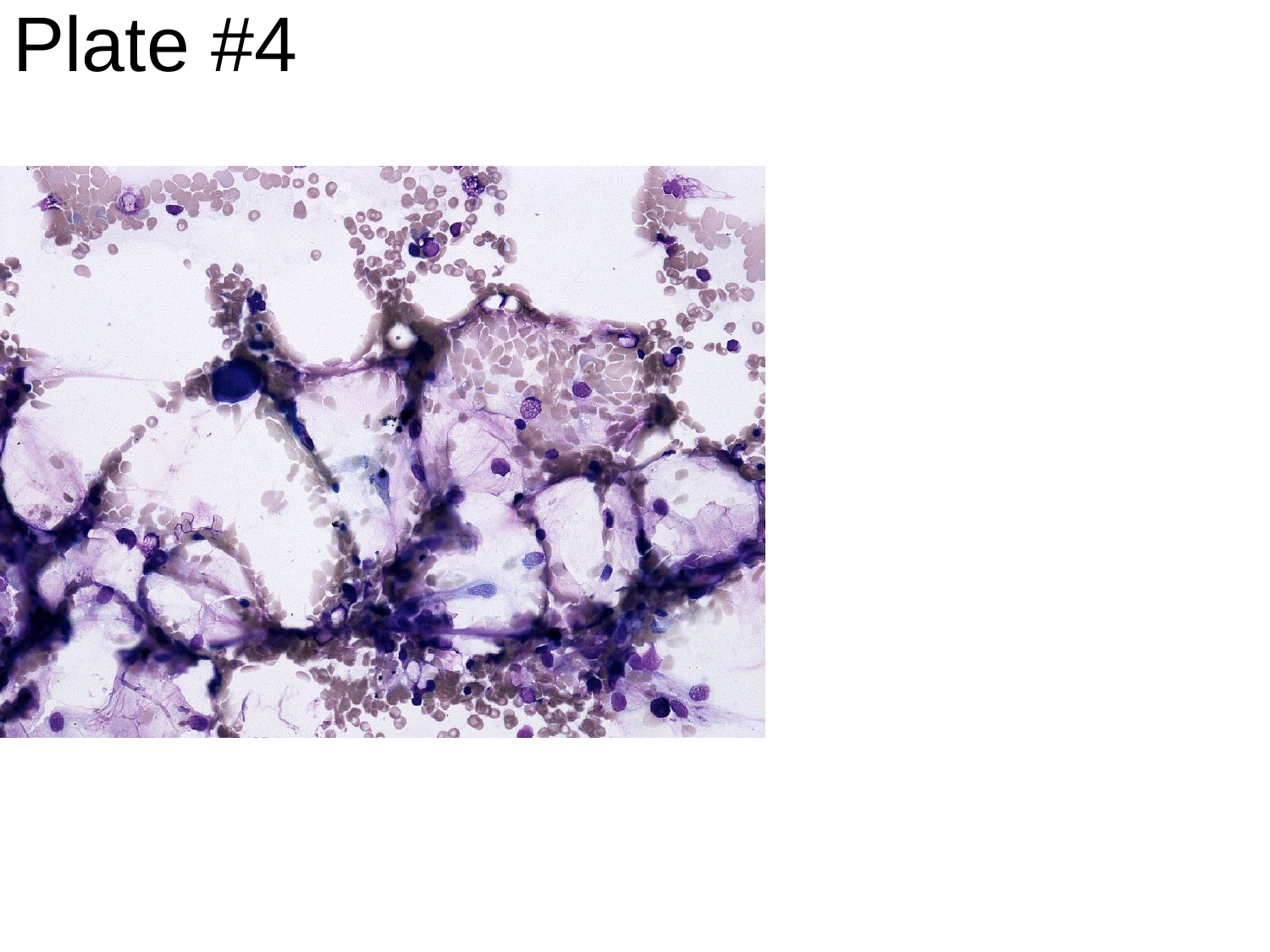

Plate #4

## Slide 5
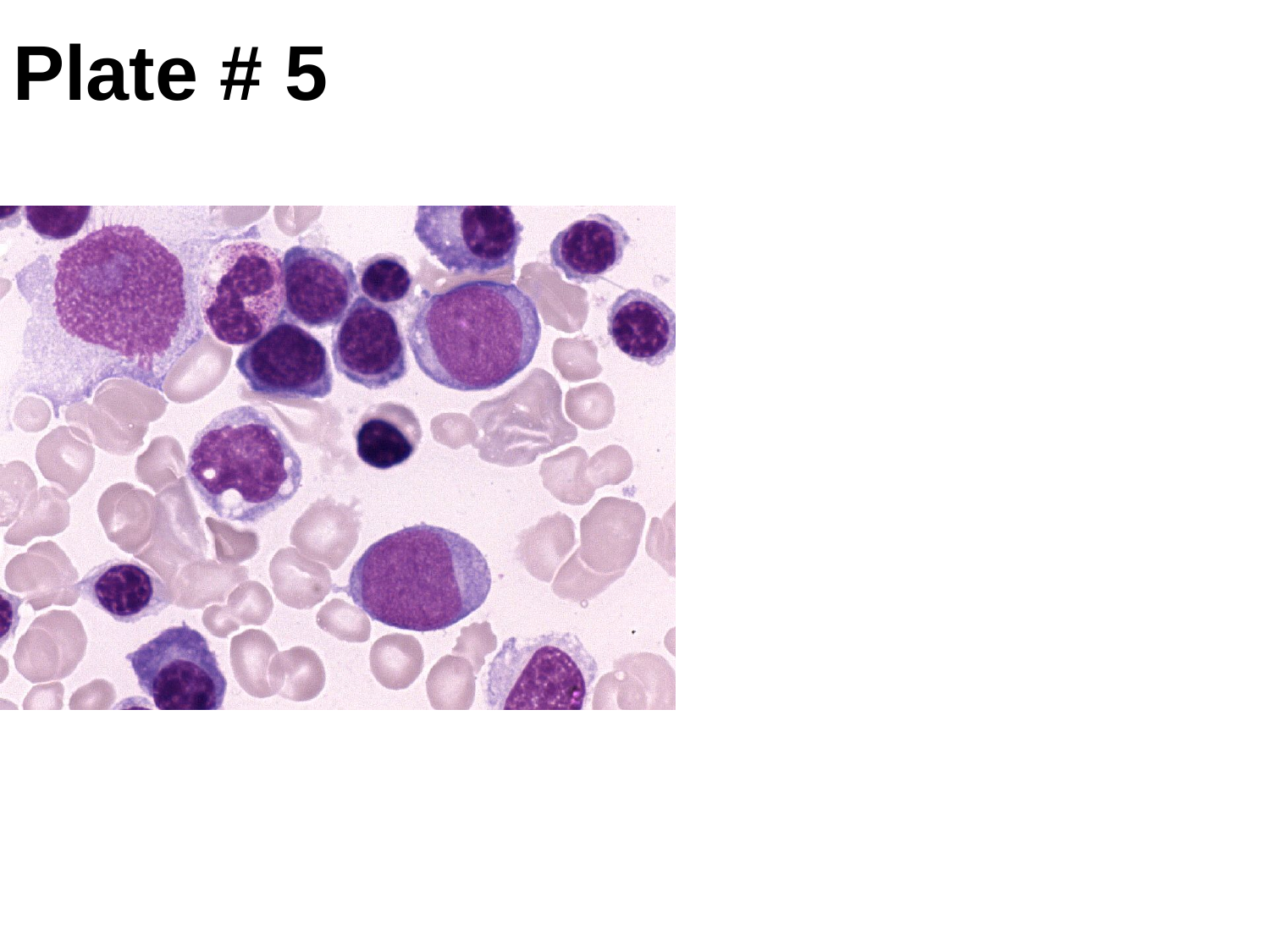

Plate # 5

## Slide 6
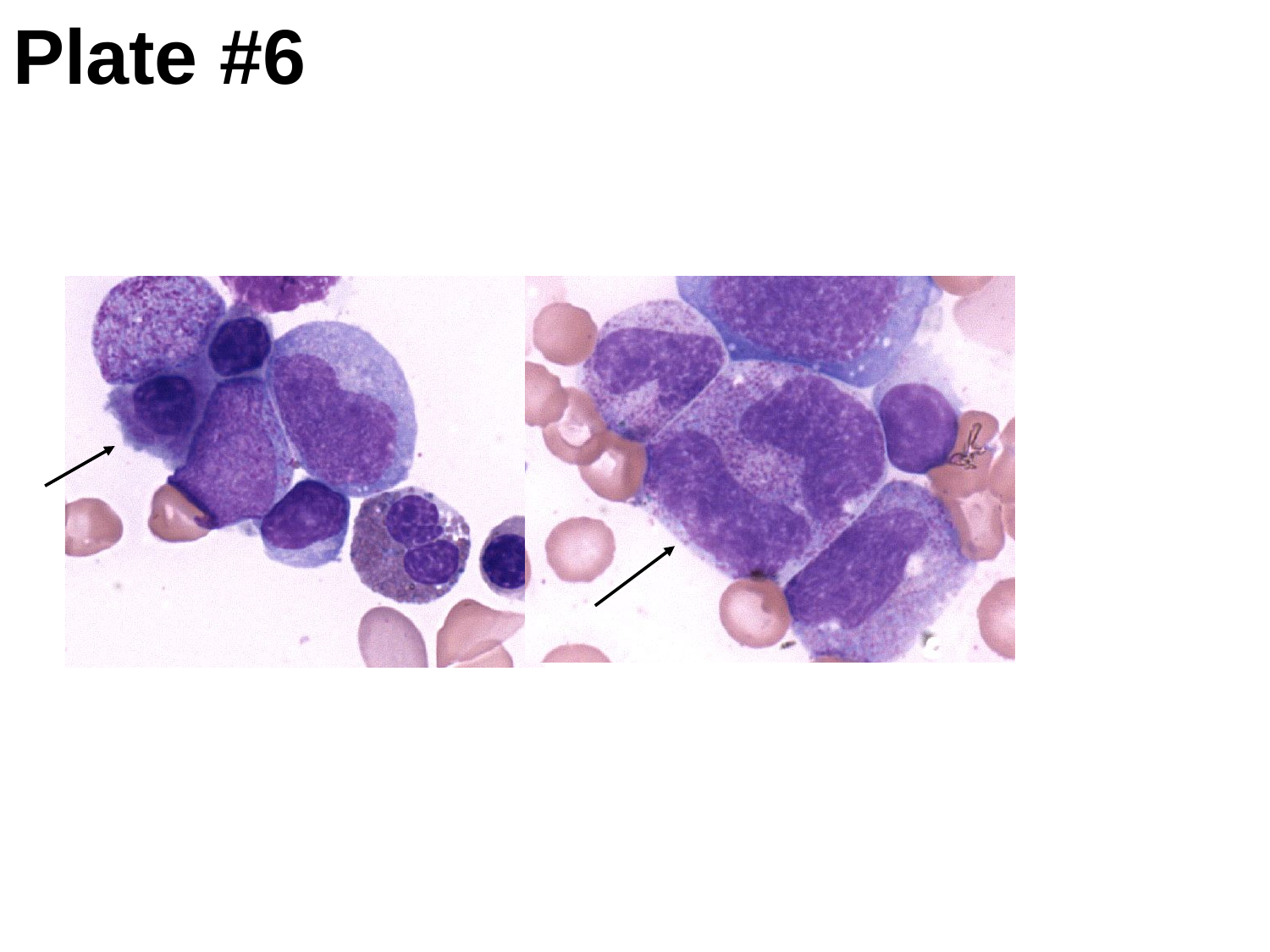

Plate #6

## Slide 7
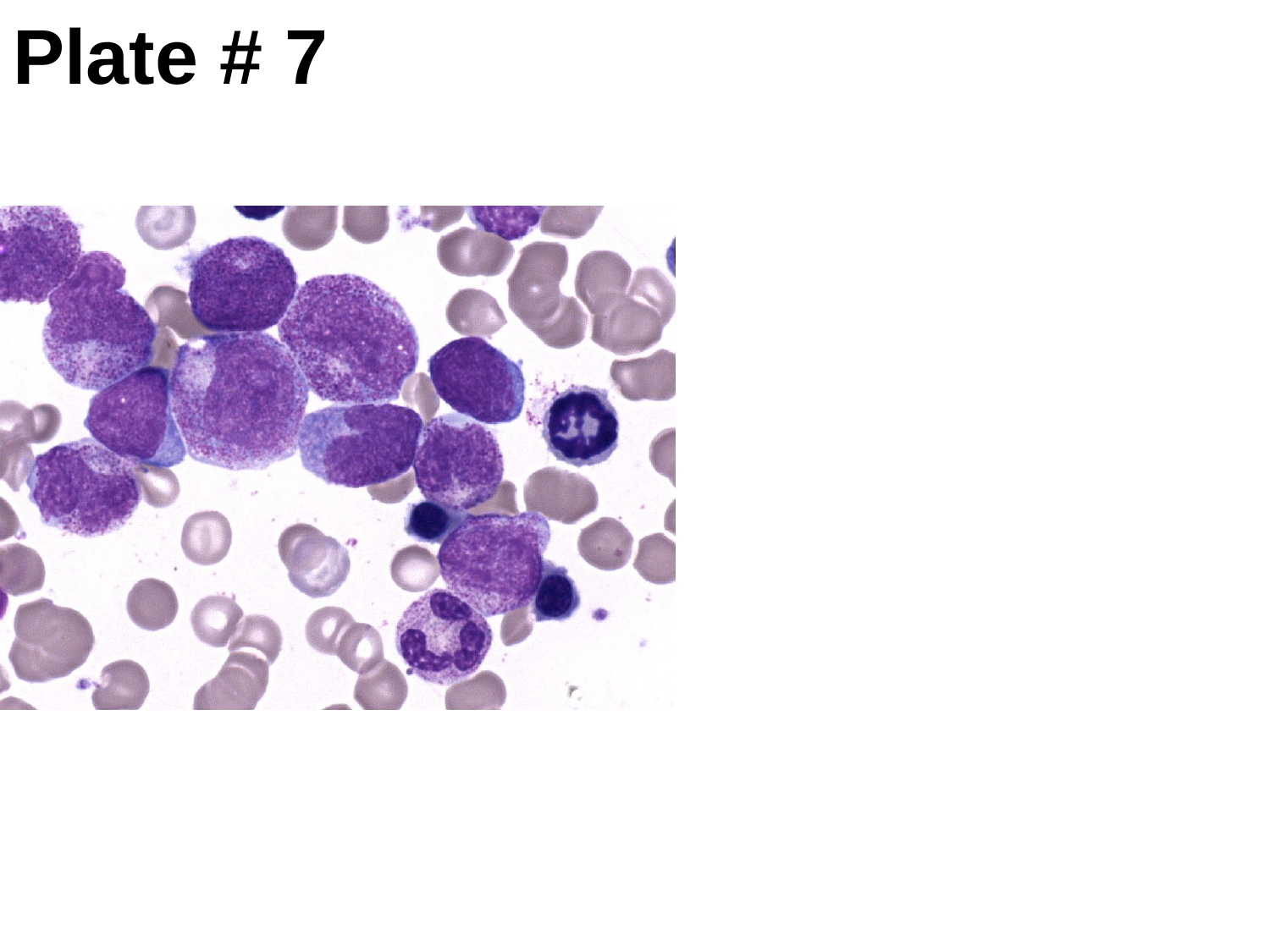

Plate # 7

## Slide 8
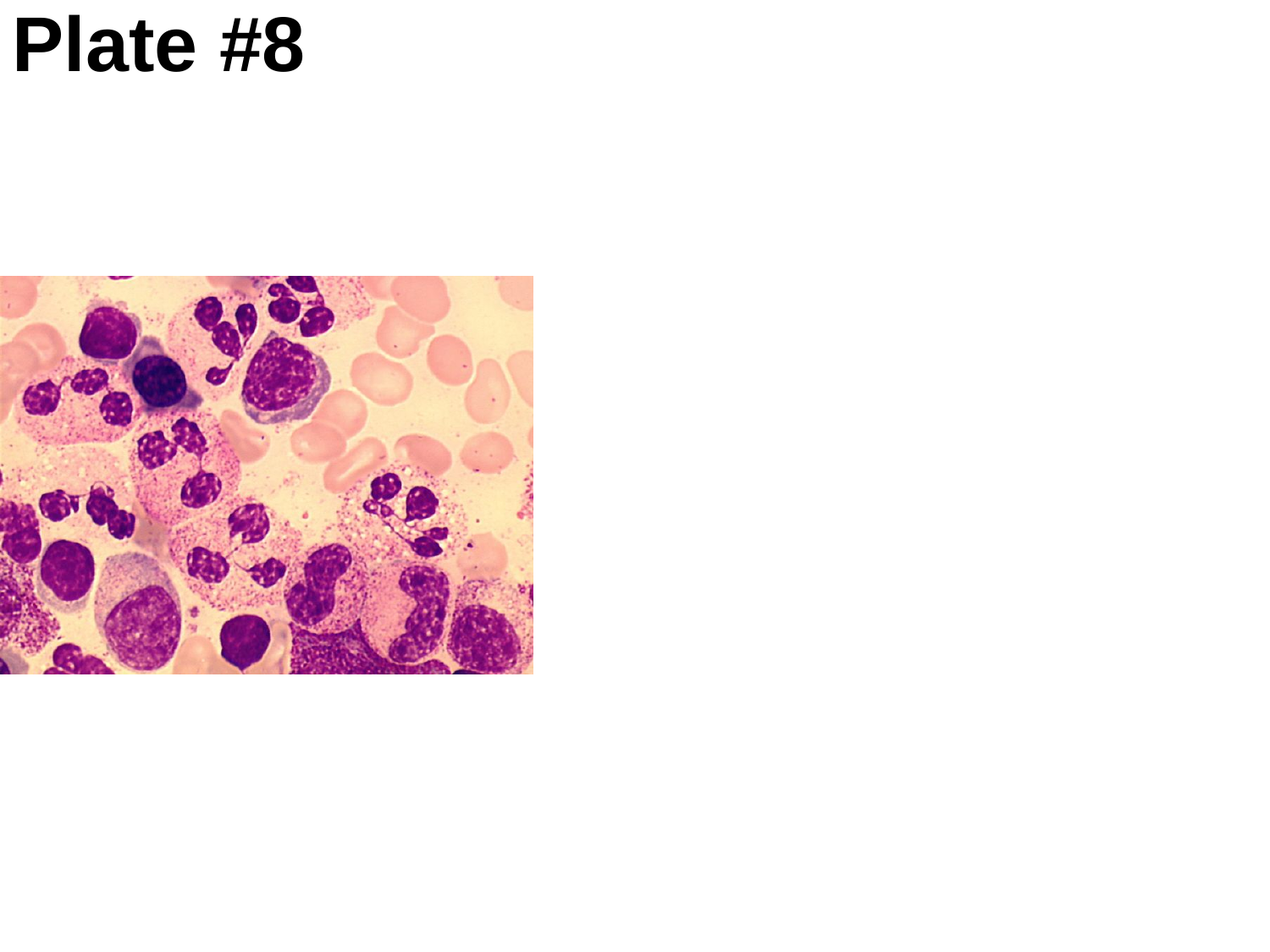

Plate #8

## Slide 9
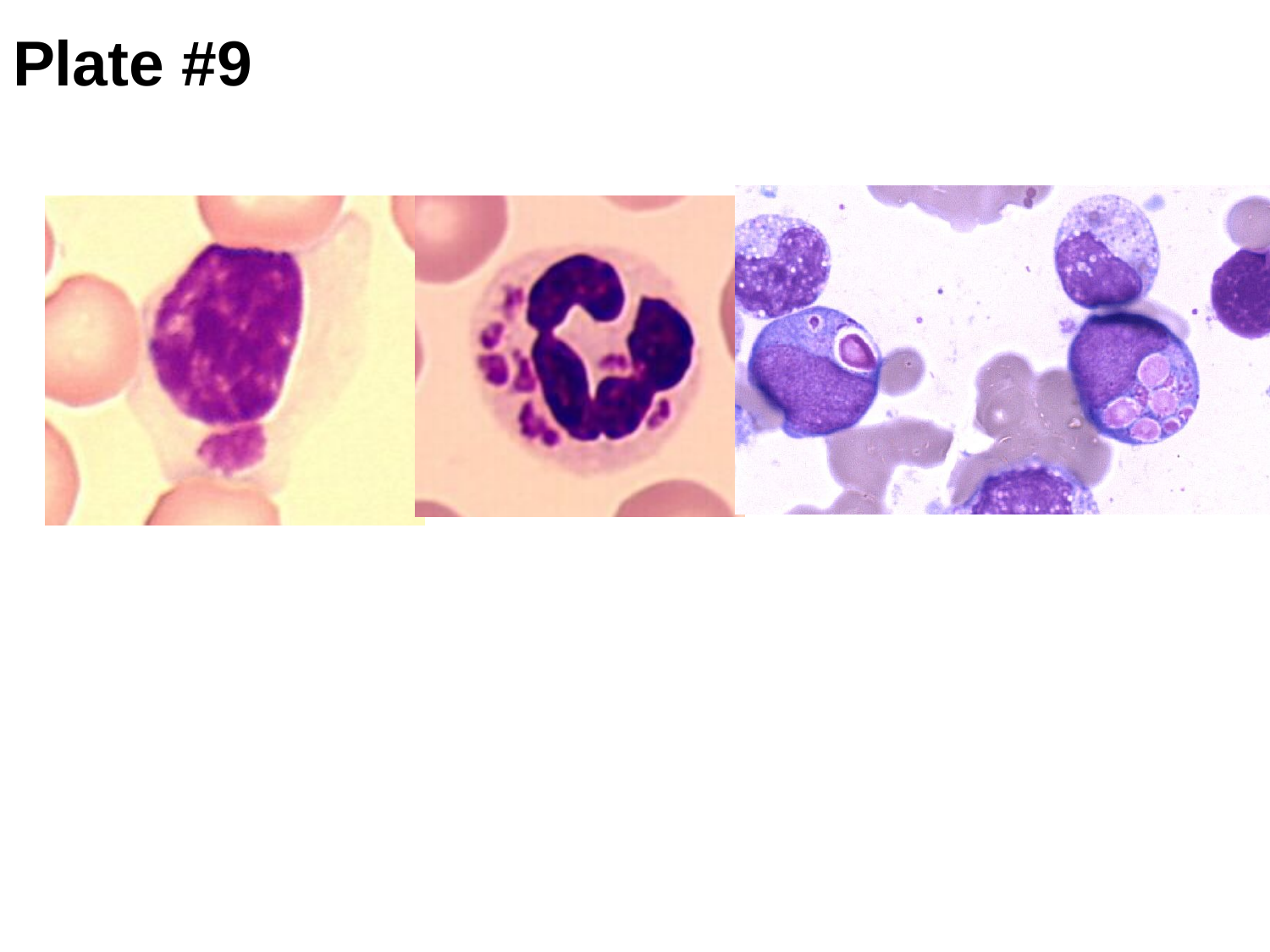

Plate #9

## Slide 10
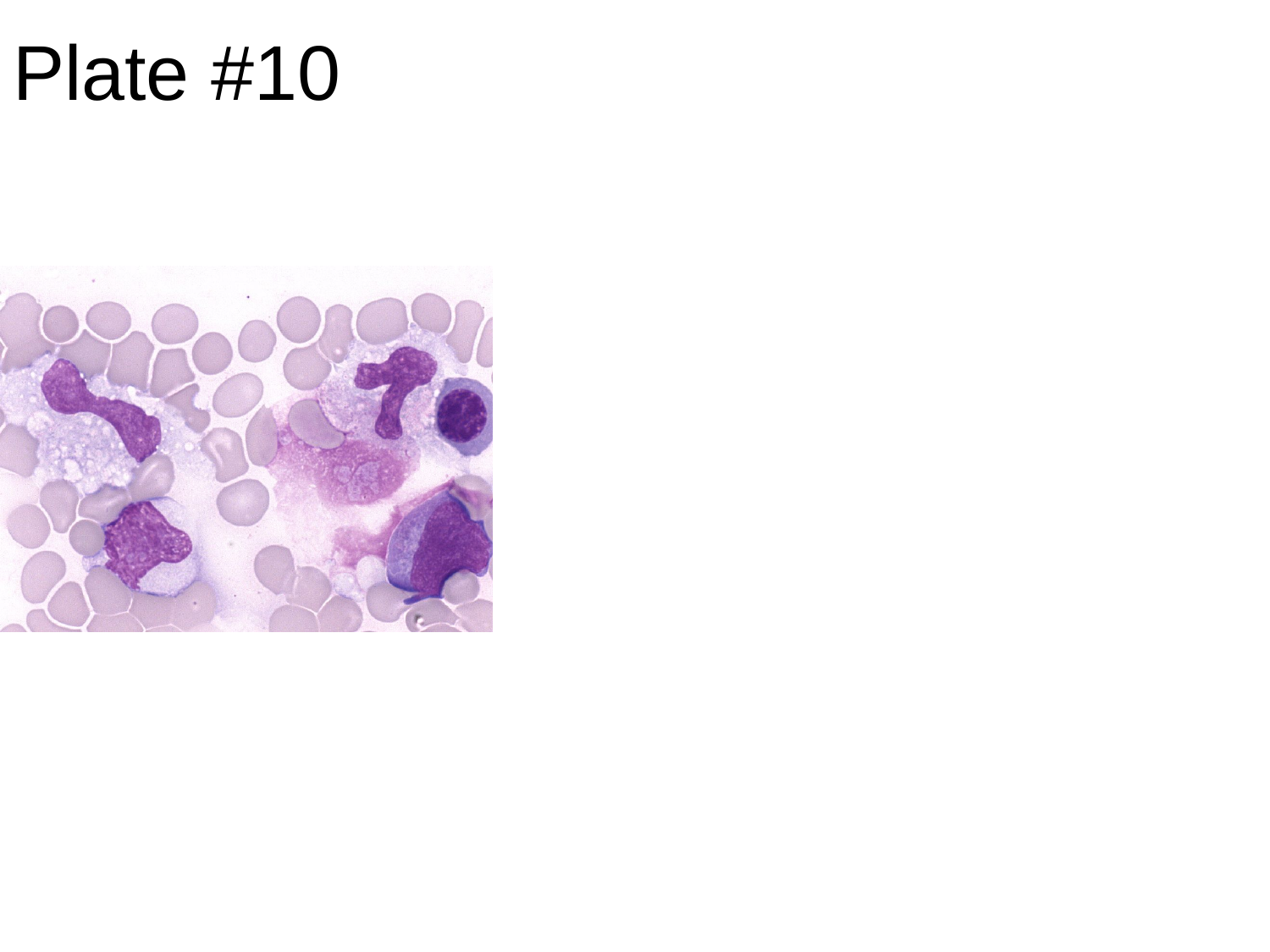

Plate #10

## Slide 11
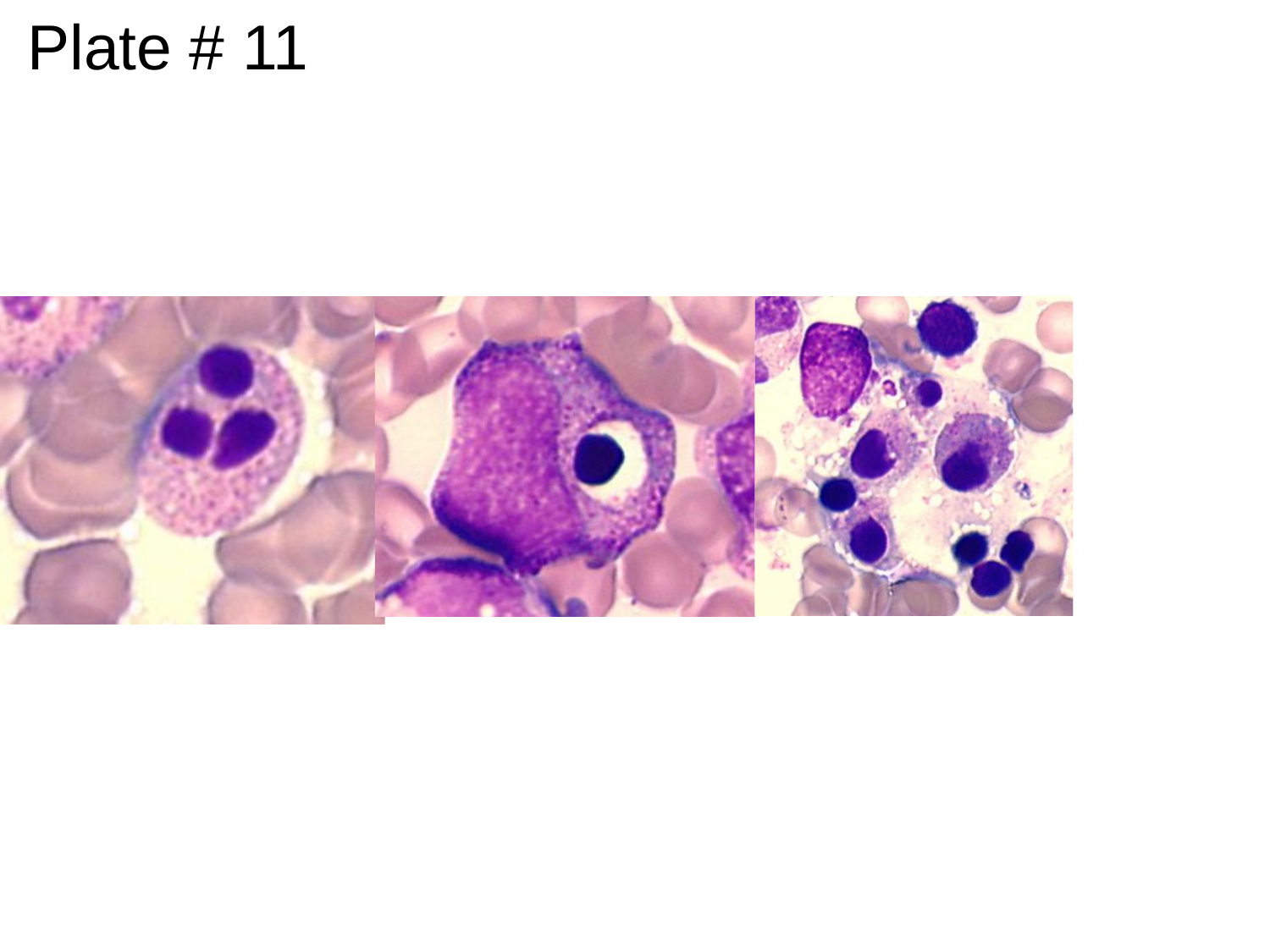

Plate # 11

## Slide 12
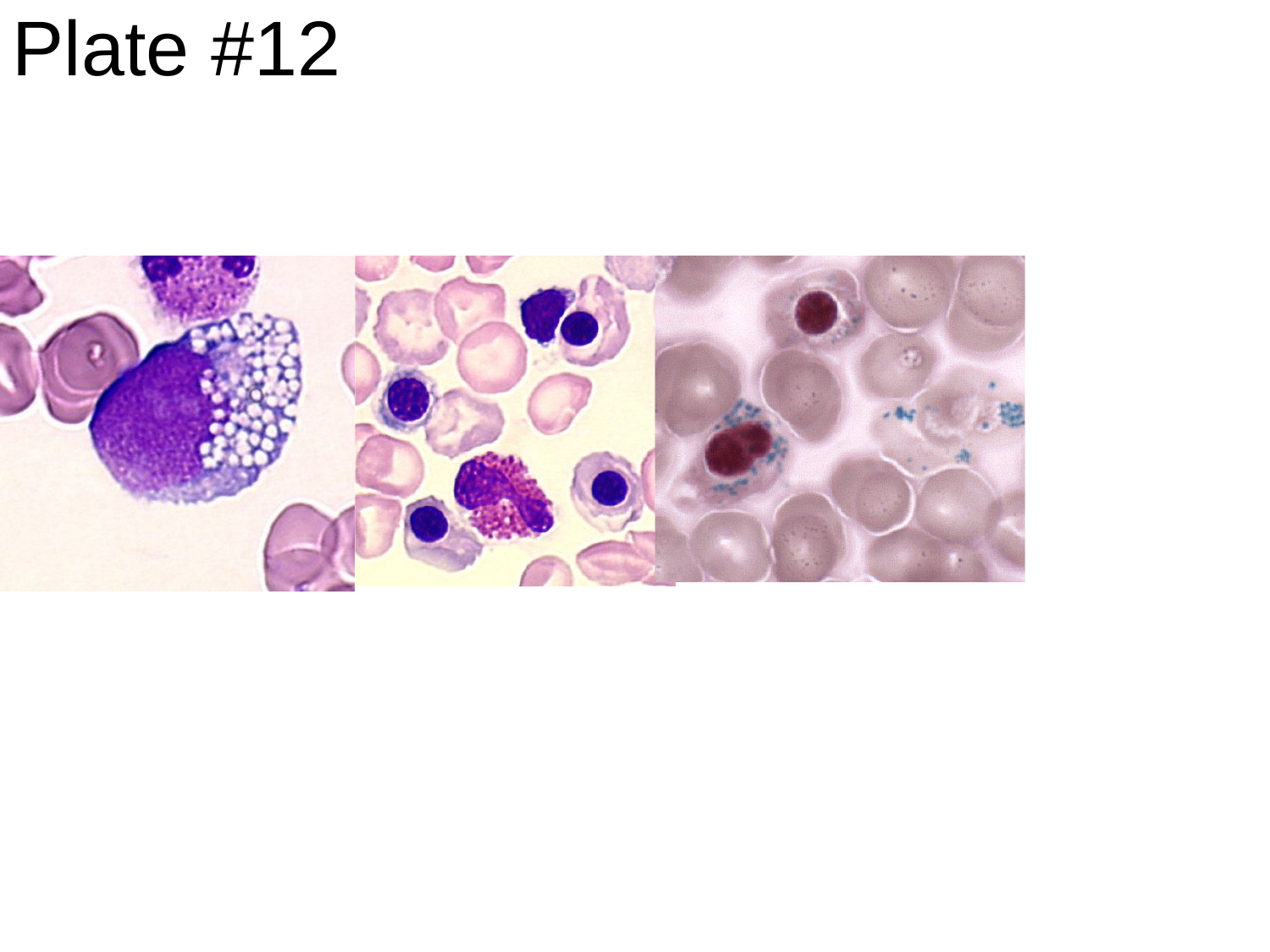

Plate #12

## Slide 13
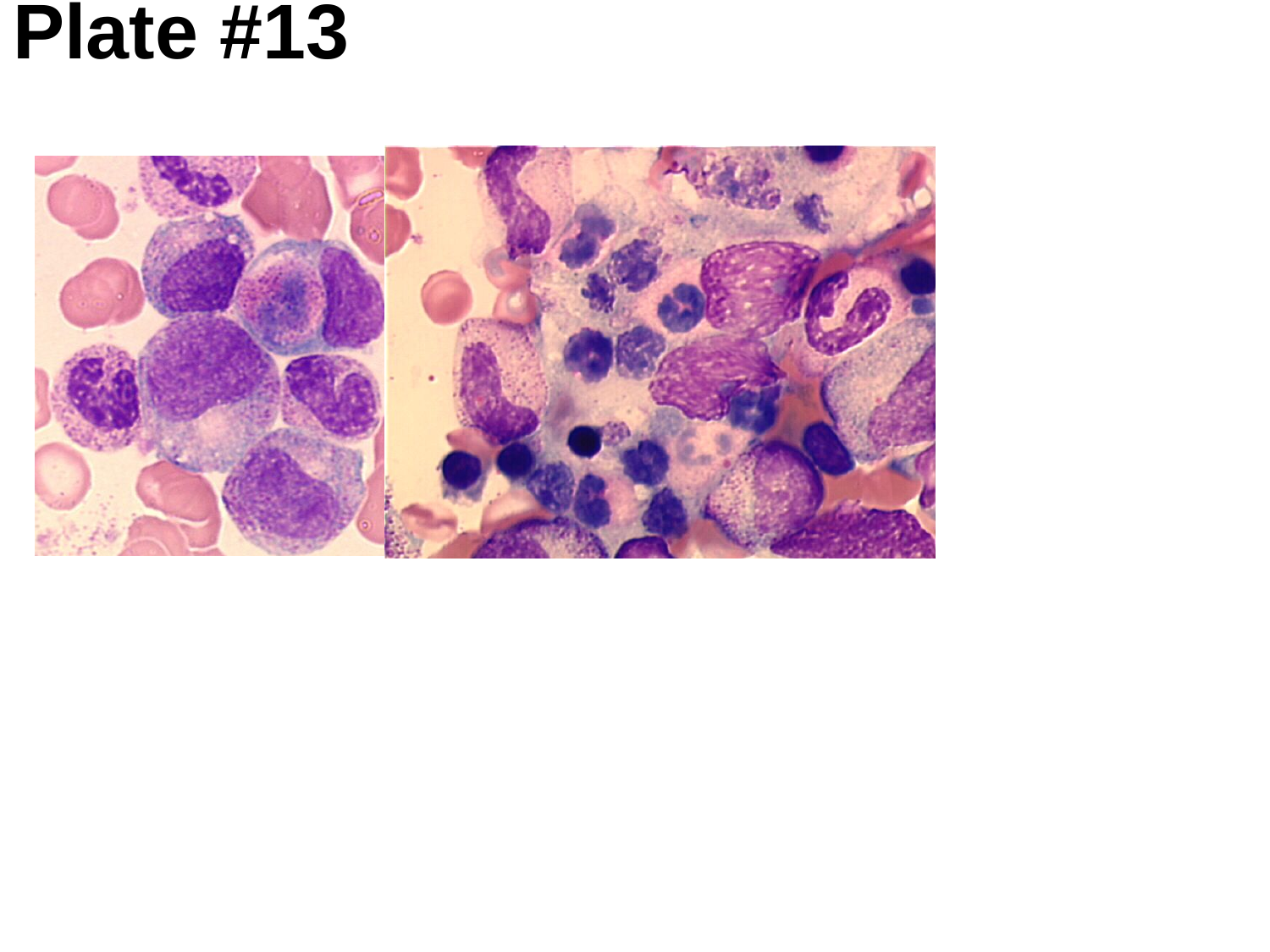

Plate #13
